# Multicomponent synthesis of α-branched amines using organozinc reagents generated from alkyl bromides

**DOI:** 10.3762/bjoc.20.239

**Published:** 2024-11-07

**Authors:** Baptiste Leroux, Alexis Beaufils, Federico Banchini, Olivier Jackowski, Alejandro Perez-Luna, Fabrice Chemla, Marc Presset, Erwan Le Gall

**Affiliations:** 1 Univ Paris-Est Créteil, CNRS, ICMPE, UMR 7182, 2 rue Henri Dunant, 94320 Thiais, France; 2 Sorbonne Université, CNRS, Institut Parisien de Chimie Moléculaire, IPCM, 75005 Paris, France

**Keywords:** alkyl bromides, branched amines, Mannich reaction, multicomponent reaction, zinc

## Abstract

The use of alkylzinc bromides in the multicomponent Mannich reaction is described. Heteroleptic organozinc compounds were obtained in THF or 2-MeTHF by direct insertion of zinc dust into the C–Br bond of alkyl bromides. It was found that the presence of a stoichiometric amount of LiCl was essential for the efficiency of the subsequent three-component coupling with aldehydes and amines. A variety of primary, secondary, and tertiary organozinc reagents as well as secondary amines and aromatic aldehydes could be used for the straightforward preparation of α-branched amines. Interestingly, whereas previously reported work describing the preparation and reaction of organozinc iodides in acetonitrile showed higher reactivity of secondary organozinc reagents over primary ones, reactions in THF in the presence of LiCl led to opposite results, with higher reactivity of primary organozinc reagents.

## Introduction

The multicomponent Mannich reaction is one of the most powerful tools available in organic synthesis for the straightforward generation of α-branched amines [1–3]. Since its discovery in 1912, the reaction has benefitted from regular improvements over the years and recent developments, such as the use of organometallic species as nucleophiles in the so-called “organometallic Mannich reaction”, which have helped to expand the boundaries of the original process [4]. In this context, while significant contributions have highlighted the reliable use of diverse organometallic species in the three-component coupling, most examples of sp^3^-hybridized compounds have remained restricted to allyl [5] or benzyl [5–6] organometallic reagents. Conversely, examples of organometallic Mannich couplings involving nonstabilized organometallics are uncommon and mostly limited to dialkylzinc reagents, likely due to their commercial availability, their significant reactivity, and their functional-group tolerance [7–10]. However, the molecular diversity accessible with these reagents is rather limited. Indeed, most examples involve dimethyl- or diethylzinc compounds as more elaborated dialkylzinc reagents represent a considerable synthetic challenge. Indeed, dialkylzinc reagents are accessible essentially through nucleophilic displacement of ZnCl_2_ by Grignard reagents or organolithium reagents, thus limiting functional-group tolerance [11–13]. By contrast, heteroleptic (mixed) alkylzinc species (i.e., RZnX) are readily available and can typically be prepared from alkyl halides by direct insertion of metallic zinc into the carbon–halogen bond [14–21]. Thus, whereas Rieke et al. reported the insertion of activated zinc into alkyl bromides in THF at room temperature [16–17], Knochel et al. described the direct metalation of alkyl iodides in THF at 30 °C [18]. More recently, Knochel et al. improved their original method by the use of zinc dust in the presence of LiCl in THF for the metalation of alkyl bromides at room temperature [19]. Besides, Huo described the insertion of zinc dust into alkyl bromides at 80 °C in DMA or DMF [20].

Despite the high synthetic interest in mixed organozinc compounds, their use in the preparation of α-branched amines remains tenuous. Indeed, until recently, mixed alkylzinc species were only employed by Carretero and co-workers in related nucleophilic additions to activated imines under Cu catalysis [22]. In 2022, our group demonstrated that alkyl iodides offer a reliable source of heteroleptic organozinc compounds through the direct insertion of zinc dust into the C–I bond in acetonitrile at 50 °C [23]. Secondary as well as tertiary organozinc iodides were found to be more reactive than primary ones. Recently, Gaunt and co-workers confirmed this tendency in a related reductive multicomponent procedure involving alkyl iodides [24]. It was indeed noticed that the reactivity of primary iodides in the multicomponent carbonyl alkylative amination (CAA) reaction was quite sluggish compared to the secondary counterparts. In addition, primary alkyl bromides were found to be almost inactive in the process. Therefore, we reasoned that replacing alkyl iodides by alkyl bromides could be relevant to the field due to interesting assets such as better availability and lower cost of the parent alkyl halides.

## Results and Discussion

We investigated new conditions that would allow for the synthesis of mixed alkylzinc bromides in order to explore their consecutive use in multicomponent Mannich reactions (Table 1).

**Table 1:** Optimization of the organozinc synthesis.^a^

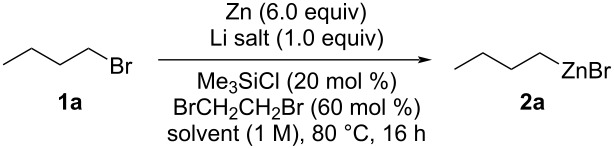			

entry	Li salt	solvent	yield^b^

1	—	CH_3_CN	0%
2	—	THF	95%
3	LiCl	THF	71%
4	LiBr	THF	10%
5	LiF	THF	52%
6	LiClO_4_	THF	0%
7	LiCl	THF^c^	73%
8	LiCl	2-MeTHF	66%

^a^Reaction conditions: Zn dust (6.0 equiv) in bulk solvent (*c* = 1.0 M) activated by chlorotrimethylsilane (20 mol %) and 1,2-dibromoethane (60 mol %), then alkyl bromide (10 mmol), 80 °C, 16 h. ^b^Titration by I_2_: A 5 mL round-bottom flask was charged with accurately weighed I_2_ (between 20 and 30 mg) and THF (2.5 mL). After the iodine was completely dissolved, the solution of organometallic reagent was added dropwise via a 1.00 mL syringe (0.01 mL graduations) until the brown color disappeared (see also [25] for details). ^c^THF was distilled over Na and benzophenone prior to use.

While our experience in the development of multicomponent reactions involving organozinc compounds prompted us to initially consider acetonitrile as probably the most adapted solvent for the whole process [26–28], we observed that this solvent was not well-suited for the preparation of the organozinc species from the corresponding bromides, with no metalation being observed after 16 h at 80 °C (Table 1, entry 1). Conversely, the organozinc compound was formed in THF in nearly quantitative yield after 16 h at 80 °C (Table 1, entry 2). However, its suitability in the multicomponent coupling with an amine and an aldehyde still had to be demonstrated as there was no precedent for such a Mannich multicomponent coupling involving nonstabilized organozinc halides using THF or 2-MeTHF as solvent. To our delight, we found that the subsequent multicomponent coupling of the organozinc bromide with piperidine and benzaldehyde was possible, although it required the additional presence of lithium chloride to furnish a satisfactory result. We attributed the beneficial role of LiCl to the formation of more nucleophilic organozincate complexes (e.g., LiBuZnBrCl), which is a well-established process in THF [29–30]. Due to the hygroscopic character of LiCl and the necessity to determine the amount of organozinc by iodolysis [20] in order to adjust the stoichiometry of the reagents for the multicomponent coupling step, this salt was more conveniently introduced to the medium at the stage of organozinc preparation. We observed that the presence of LiCl was not deleterious for the metalation, although the organozinc yield decreased to 71% (Table 1, entry 3), probably due to the initial presence of water traces in the reaction medium. With these results in hands, we tried to determine whether LiCl could be replaced by other common lithium salts that could lead to improved metalation (Table 1, entries 4–6). These experiments revealed that LiCl gave the best results, as a significant drop of the zincation yield was obtained with both LiBr (Table 1, entry 4) and LiClO_4_ (Table 1, entry 6), whereas LiF gave an acceptable yield of organozinc reagent (Table 1, entry 5). Therefore, additional experiments were carried out with LiCl. We first tried to determine whether the use of distilled (Table 1, entry 7) instead of commercial THF (Table 1, entry 3) could have a significant impact on the zincation step and observed that this was not the case, with a comparable yield obtained under both conditions. Finally, we evaluated the influence of another ethereal solvent on the metalation and, notably, found that the use of biosourced 2-MeTHF led to a comparable result (Table 1, entry 8).

**Scheme 1:**
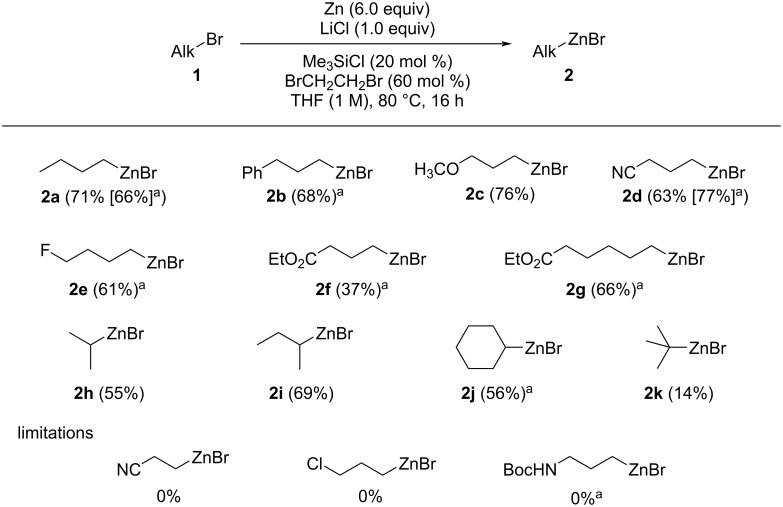
Scope of organozinc reagents. Yield was determined by titration with I_2_. Reaction conditions: Zn dust (6.0 equiv) in bulk THF (*c* = 1.0 M) activated by chlorotrimethylsilane (20 mol %) and 1,2-dibromoethane (60 mol %), then alkyl bromide (10 mmol), 80 °C, 16 h. ^a^2-MeTHF was used as solvent.

With these results in hands, we evaluated the scope of the zincation. The results are presented in Scheme 1.

**Scheme 2:**
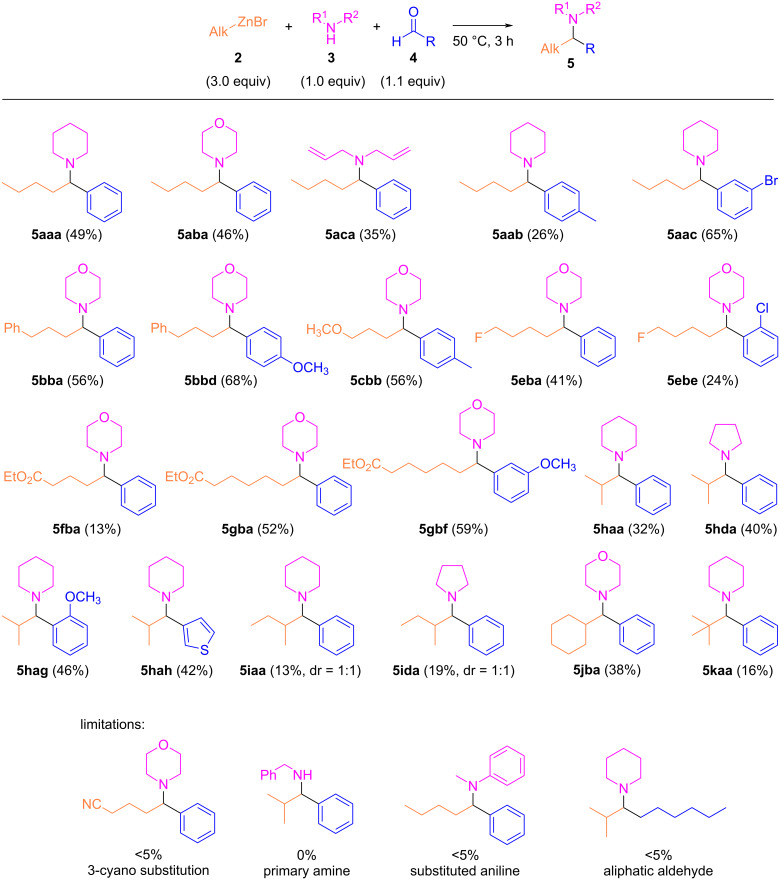
Scope of the reaction. Yield of isolated product is given.

The zincation conditions proved to be general, with a good yield of the organozinc compounds being obtained after 16 h at 80 °C in THF. It could be noted that comparable results were obtained in both THF and 2-MeTHF (i.e., organozinc compounds **2a** and **2d**). Interestingly, whereas simple primary (i.e., compounds **2a**–**g**) and secondary (i.e., compounds **2h**–**j**) aliphatic organozinc species could be obtained efficiently, a tertiary bromide furnished deceiving zincation results (i.e., compound **2k**). However, we were delighted to notice that functionalized organozinc species could be accessed from the corresponding bromides (i.e., organozinc compounds **2c**–**g**). After reaction and centrifugation, the solutions of the organozinc species **2** were collected using a syringe and allowed to react with an amine **3** and an aldehyde **4** under moderate heating (Scheme 2).

The multicomponent couplings proceeded smoothly, with an acceptable yield of the α-branched amine being obtained after 3 h at 50 °C. A range of secondary amines as well as various aromatic aldehydes could be used in the multicomponent coupling. Interestingly, functionalized organozinc reagents gave the multicomponent coupling product, except for the cyanated organozinc compound **2d**, which only provided traces of the expected α-branched amine. It can be noted that in sharp contrast to our previous reports on the related multicomponent reaction involving organozinc iodides in acetonitrile, for which secondary organozinc compounds provided better results than primary ones, primary organozinc bromides reacted more efficiently than secondary ones in THF in the presence of LiCl. In addition, whereas *tert*-butylzinc iodide was efficient in the multicomponent coupling (99% yield), *tert*-butylzinc bromide led to a very low yield (16% **5kaa**). The coupling presented some other limitations. It was not possible to carry out the reaction when a primary amine was employed. In addition, a secondary aniline only furnished traces of the expected product. Aliphatic aldehydes also failed to deliver the multicomponent adduct.

## Conclusion

In conclusion, we have shown in this work that alkyl bromides can be used instead of alkyl iodides in a direct zincation–multicomponent organometallic Mannich reaction sequence to furnish α-branched amines in an acceptable yield. The reaction sequence is conducted in THF or 2-MeTHF, and the presence of LiCl is essential. Although the scope of the reaction is narrower than the analogous process relying on organozinc iodides, the reaction offers significant assets associated to the use of alkyl bromides, which are more easily prepared (or commercially available), less costly, and more stable than the corresponding alkyl iodides. In addition, the reaction can be conducted in commercial THF or 2-MeTHF and without special precautions. We also found a reversed reactivity order of primary and secondary organozinc bromides in comparison to that of organozinc iodides. Herein, primary organozinc compounds reacted better than secondary ones.

## Supporting Information

File 1 Experimental procedures, compound characterization data, and NMR spectra for all compounds.

## Data Availability

All data that supports the findings of this study is available in the published article and/or the supporting information of this article.
