# Supplementary material for: Multicomponent synthesis of α-branched amines using organozinc reagents generated from alkyl bromides
Source: Beilstein J Org Chem. 2024 Nov 7;20:2834–9. doi: 10.3762/bjoc.20.239 (PMC11552408; doi:10.3762/bjoc.20.239)

BEILSTEIN JOURNAL OF ORGANIC CHEMISTRY

Supporting Information

for

Multicomponent synthesis of α-branched amines using organozinc reagents generated from alkyl bromides

Baptiste Leroux, Alexis Beaufils, Federico Banchini, Olivier Jackowski, Alejandro Perez-Luna, Fabrice Chemla, Marc Presset and Erwan Le Gall

Beilstein J. Org. Chem. **2024,** *20,* 2834–2839. doi:10.3762/bjoc.20.239

Experimental procedures, compound characterization data, and NMR spectra for all compounds

License and Terms: This is a supporting information file under the terms of the Creative Commons Attribution License (https://creativecommons.org/licenses/by/4.0). Please note that the reuse, redistribution and reproduction in particular requires that the author(s) and source are credited and that individual graphics may be subject to special legal provisions.
The license is subject to the *Beilstein Journal of Organic Chemistry* terms and conditions: (https://www.beilstein-journals.org/bjoc/terms)

Table of contents

1. General information S2
2. Prices/availability of aliphatic iodides vs bromides S2
3. Formation and reactivity of organozinc reagents generated in THF or MeCN S3
4. Organozinc reagents S4
5. Mannich reactions S5
6. Copies of NMR spectra S10

1. General information

• All commercially available reagents, including solvents (tetrahydrofuran THF, 2-methyltetrahydrofuran = 2-MeTHF, cyclohexane = Cy, ethyl acetate = EA), were used as received.

• Room temperature means 18 – 25 °C.

• Analytical thin layer chromatography (TLC) was performed on TLC silica gel plates (0.25 mm) precoated with a fluorescent indicator. Visualization was effected using ultraviolet light (λ = 254 nm) and/or a aqueous solution of $KMnO_4$. Flash chromatography (FC) was performed on 40–63 µm silica gel with mixtures of solvents.

• High-resolution mass spectra were obtained at the ICOA of the Université of Orléans by electrospray ionization using a Q-TOF analyzer.

• NMR spectra were recorded on a 400 MHz spectrometer. ^{1}H NMR chemical shifts were referenced to the residual solvent signal; ^{13}C NMR chemical shifts were referenced to the deuterated solvent signal. Multiplicity was defined by DEPT 135 analysis. Data are presented as follows: chemical shift δ (ppm), multiplicity (*s* = singlet, *d* = doublet, *t* = triplet, *q* = quartet, *m* = multiplet, *br* = broad), coupling constant *J* (Hz), integration.

• dr was determined by NMR analysis of the crude reaction mixture.

• Compounds **5aaa**,[1] **5aba**,[1] **5fba**,[2] **5haa**,[1] **5hda**,[1] **5hag**,[1] **5hah**,[1] **5iaa**[1] and **5kaa**[1] have been previously described.

2. Prices/availability of aliphatic iodides vs bromides

Prices (France) for common aliphatic iodides and bromides, reported from the Sigma-Aldrich web site (https://www.sigmaaldrich.com/FR/fr).

Compound	Amount	Price	Quality	Stabilization
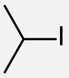	100 g	51.00 €	99%	Copper
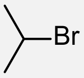	100 g	28.00 €	99%	
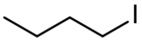	100 mL	76.30 €	For synthesis	
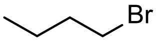	100 mL	27.10 €	For synthesis	
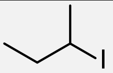	100 g	343.00 €	98%+	Copper
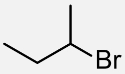	100 g	38.40 €	98%	
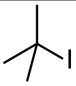	100 g	356.00 €	95%	Copper
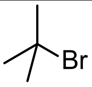	100 g	86.10 €	98%	
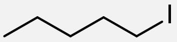	100 g	71.30 €	98%	
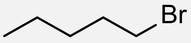	100 g	28.00 €	98%	
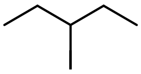	Not available	-	-	
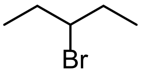	25 g	183.00 €	95%	
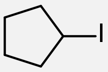	25 mL (~42 g)	444.00 €	97%	

[1] M. Pinaud, E. Le Gall, M. Presset, *J. Org. Chem.* **2022**, *87*, 4961–4964.
[2] T. Hou, P. Lu, P. Li, *Tetrahedron Lett.* **2016**, *57*, 2273–2276.

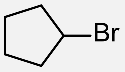	25 mL	37.00 €	98%+	
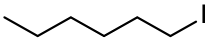	100 g	75.00 €	98%+	
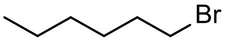	100 g	23.10 €	98%	
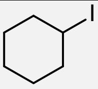	100 g	249.00 €	98%	
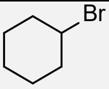	100 g	31.20 €	98%	
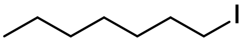	50 g	52.80 €	98%	
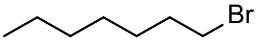	100 g	53.40 €	99%	
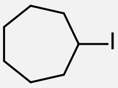	Not available	-	-	
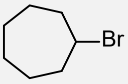	25 g	92.80 €	97%	

3. Formation and reactivity of organozinc reagents generated in THF or MeCN

Comparison of the preparation and reactivity in the Mannich reaction of organozinc compounds originating from primary alkyl bromides (reaction in THF described herein) and alkyl iodides (reaction in MeCN as described in JOC 2022).

Entry	Alkyl halide	Organozinc yield (iodolysis)	Mannich adduct	Yield
1	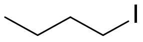	19%	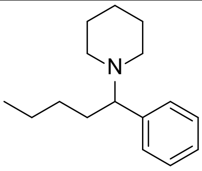	NR
2	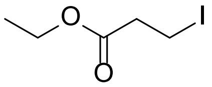	-	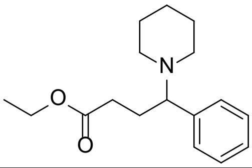	NR
3	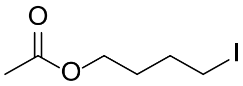	62%	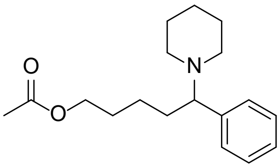	NR
4	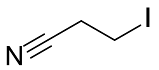	99%	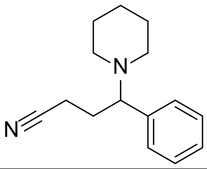	NR
5	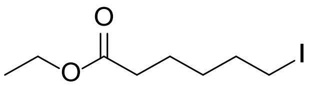	60%	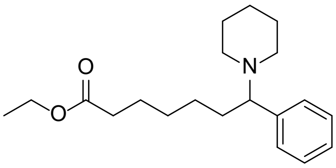	NR

6	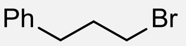	68%	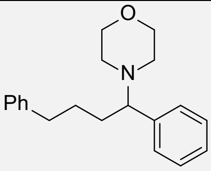	56%
7	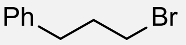	68%	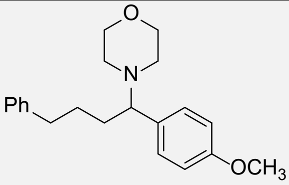	68%
8	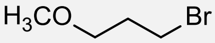	76%	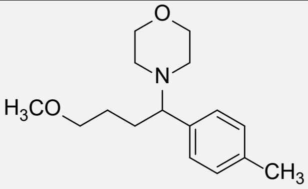	56%
9	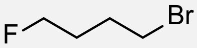	61%	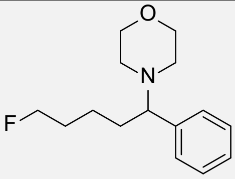	41%
10	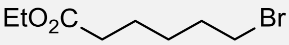	66%	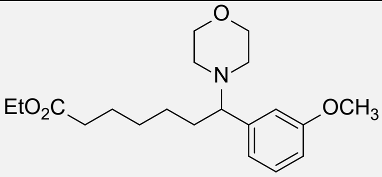	59%

4. Organozinc reagents

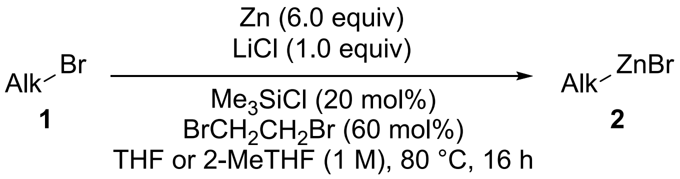

In air, a 30 mL reaction tube equipped with a stir bar was charged with zinc dust (3.92 g, 60 mmol, 6.0 equiv) and LiCl (0.42 g, 10 mmol, 1.0 equiv) and purged with Ar. THF or 2-MeTHF (10 mL, C = 0.5 M) was added and the resulting suspension was treated with chlorotrimethylsilane (0.25 mL, 2 mmol, 20 mol %) and stirred at room temperature for 2 min. 1,2-dibromoethane (0.52 mL, 6 mmol, 60 mol %) was added dropwise and the resulting suspension was at room temperature for 5 min. The alkyl bromide 1 (10 mmol, 1.0 equiv) was added and the reaction was stirred at 80 °C for 16 h. After cooling to room temperature, the solution was centrifugated at 3500 rpm for 15 min. The concentration of the solution of organozinc reagent was determined by iodometric titration of the resulting supernatant.

**Organozinc reagent**	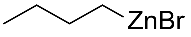	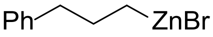	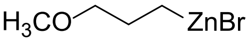	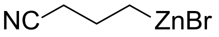
**Yield in THF (%)**	71	-	76	63
**Yield in 2-MeTHF (%)**	66	68	-	77
**Organozinc reagent**	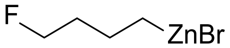	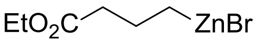	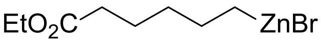	
**Yield in THF (%)**	-	-	-	
**Yield in 2-MeTHF (%)**	61	37	66	

Organozinc reagent	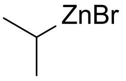	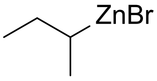	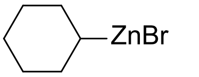	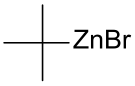
Yield in THF (%)	55	69	-	14
Yield in 2-MeTHF (%)	-		56	-

5. Mannich reactions

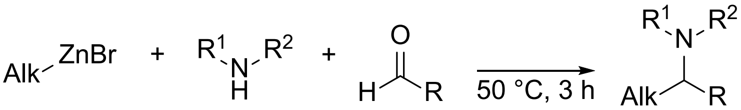

2 (3.0 equiv) **3** (1.0 equiv) **4** (1.1 equiv) **5**

General procedure (GP): A 30 mL reaction tube equipped with a stir bar and filled with an Ar atmosphere was charged with aldehyde (1.1 equiv) and amine (1.0 equiv). A solution of the organozinc reagent (3.0 equiv) was added and the reaction was stirred at 50 °C for 3 h. Then, the reaction mixture was cooled down to room temperature and poured into sat aq NH_4Cl. The resulting solution was extracted twice with EtOAc and the combined organic layers were washed brine, dried over anhydrous Na_2SO_4 and evaporated. The crude product was purified by FC to afford the desired product.

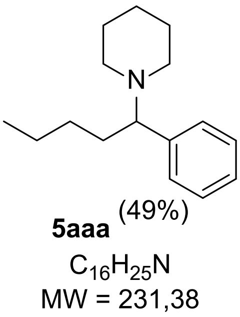

5aaa (49%)
$C_{16}H_{25}N$
MW = 231,38

Compound **5aaa**:[1] Following the **GP** performed with piperidine (73 µL, 0.73 mmol, 1.0 equiv), benzaldehyde (82 µL, 0.81 mmol, 1.1 equiv) and a solution of alkylkzinc bromide in THF (3.5 mL, 0.63 M, 2.20 mmol, 3.0 equiv), the desired product was obtained after purification by FC [V(SiO_2) = 25 mL, Cy/EA : 9/1 (100 mL), 8/2 (150 mL)] as a pale yellow oil (83 mg, 49%).

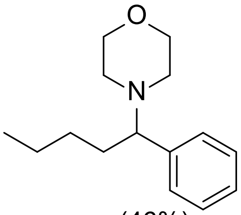

5aba (46%)
$C_{15}H_{23}NO$
MW = 233,36

Compound **5aba**:[1] Following the **GP** performed with morpholine (67 µL, 0.77 mmol, 1.0 equiv), benzaldehyde (86 µL, 0.85 mmol, 1.1 equiv) and a solution of alkylkzinc bromide in 2-MeTHF (3.5 mL, 0.66 M, 2.31 mmol, 3.0 equiv), the desired product was obtained after purification by FC [V(SiO_2) = 25 mL, Cy/EA : 9/1 (150 mL), 8/2 (100 mL)] as a colorless oil (82 mg, 46%).

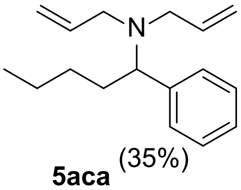

5aca (35%)
$C_{17}H_{25}N$
MW = 243,39

Compound **5aca**: Following the **GP** performed with diallylamine (75 µL, 0.61 mmol, 1.0 equiv), benzaldehyde (68 µL, 0.67 mmol, 1.1 equiv) and a solution of alkylkzinc bromide in THF (3 mL, 0.61 M, 1.82 mmol, 3.0 equiv), the desired product was obtained after purification by FC [V(SiO_2) = 25 mL, Cy/EA : 9.5/0.5 (150 mL), 9/1 (100 mL)] as a colorless oil (52 mg, 35%).

R_f (SiO_2, Cy/EA : 9/1): 0.63.
HRMS (ESI+): m/z $[M+H]^+$ Calcd. for $C_{17}H_{26}N$: 244.2060. Found: 244.2063.

^{1}H NMR (400 MHz, CDCl$_3$): δ 7.41 – 7.37 (m, 2H), 7.33 – 7.29 (m, 3H), 5.95 – 5.85 (m, 2H), 5.25 – 5.17 (m, 4H), 3.78 (dd, *J* = 8.6, 6.1 Hz, 1H), 3.35 (dd, *J* = 14.4, 4.9 Hz, 2H), 2.85 (dd, *J* = 14.4, 7.5 Hz, 2H), 2.01 – 1.93 (m, 1H), 1.86 – 1.78 (m, 1H), 1.40 – 1.30 (m, 3H), 1.24 – 1.16 (m, 1H), 0.93 (t, *J* = 7.1 Hz, 3H).
^{13}C{^{1}H} NMR (100 MHz, CDCl$_3$): δ 140.5 (C), 137.2 (2 CH), 128.9 (2 CH), 128.0 (2 CH), 126.9 (CH), 116.8 (2 CH$_2$), 63.5 (CH), 52.8 (2 CH$_2$), 32.1 (CH$_2$), 29.1 (CH$_2$), 22.9 (CH$_2$), 14.2 (CH$_3$).

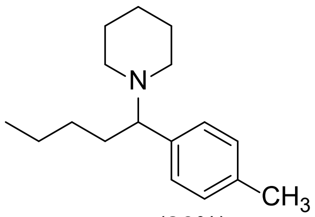

5aab (26%)
$C_{17}H_{27}N$
MW = 245,41

Compound **5aab**: Following the **GP** performed with piperidine (60 µL, 0.61 mmol, 1.0 equiv), *p*-tolualdehyde (79 µL, 0.67 mmol, 1.1 equiv) and a solution of alkylkzinc bromide in THF (3 mL, 0.61 M, 1.82 mmol, 3.0 equiv), the desired product was obtained after purification by FC [V(SiO$_2$) = 25 mL, Cy/EA : 9/1 (150 mL), 8/2 (100 mL)] as a colorless oil (39 mg, 26%).

R*f* (SiO$_2$, Cy/EA : 9/1): 0.08.
HRMS (ESI+): m/z [M+H]$^+$ Calcd. for $C_{17}H_{28}N$: 246.2216. Found: 246.2220.
^{1}H NMR (400 MHz, CDCl$_3$): δ 7.13 (d, *J* = 8.0 Hz, 2H), 7.09 (d, *J* = 8.0 Hz, 2H), 3.29 (dd, *J* = 9.5, 5.0 Hz, 1H), 2.35 (br s, 7H), 1.91 – 1.75 (m, 2H), 1.60 – 1.48 (m, 4H), 1.39 – 1.22 (m, 4H), 1.15 – 1.00 (m, 2H), 0.83 (t, *J* = 7.3 Hz, 3H).
^{13}C{^{1}H} NMR (100 MHz, CDCl$_3$): δ 134.0 (C), 136.3 (C), 128.96 (2 CH), 128.6 (2 CH), 70.4 (CH), 51.3 (2 CH$_2$), 32.6 (CH$_2$), 29.1 (CH$_2$), 26.5 (2 CH$_2$), 24.8 (CH$_2$), 23.0 (CH$_2$), 21.2 (CH$_3$), 14.2 (CH$_3$).

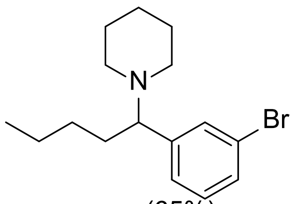

5aac (65%)
$C_{16}H_{24}BrN$
MW = 310,28

Compound **5aac**: Following the **GP** performed with piperidine (73 µL, 0.73 mmol, 1.0 equiv), 3-bromobenzaldehyde (94 µL, 0.81 mmol, 1.1 equiv) and a solution of alkylkzinc bromide in THF (3.5 mL, 0.63 M, 2.20 mmol, 3.0 equiv), the desired product was obtained after purification by FC [V(SiO$_2$) = 25 mL, Cy/EA : 9/1 (150 mL), 8/2 (100 mL)] as a yellow oil (149 mg, 65%).

R*f* (SiO$_2$, Cy/EA : 8/2): 0.51.
HRMS (ESI+): m/z [M+H]$^+$ Calcd. for $C_{16}H_{25}BrN$: 310.1165. Found:310.1163.
^{1}H NMR (400 MHz, CDCl$_3$): δ 7.42 – 7.40 (m, 2H), 7.23 – 7.16 (m, 2H), 3.29 (dd, *J* = 9.4, 4.9 Hz, 1H), 2.36 (br s, 4H), 1.92 – 1.85 (m, 1H), 1.82 – 1.74 (m, 1H), 1.59 – 1.55 (m, 4H), 1.41 – 1.28 (m, 4H), 1.21 – 1.05 (m, 2H), 0.87 (t, *J* = 7.3 Hz, 3H).
^{13}C{^{1}H} NMR (100 MHz, CDCl$_3$): δ 143.1 (C), 131.6 (CH), 129.9 (CH), 129.4 (CH), 127.4 (CH), 122.2 (C), 70.2 (CH), 51.2 (2 CH$_2$), 35.3 (CH$_2$), 28.8 (CH$_2$), 26.4 (2 CH$_2$), 24.7 (CH$_2$), 22.9 (CH$_2$), 14.1 (CH$_3$).

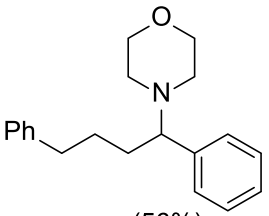

5bba (56%)
$C_{20}H_{25}NO$
MW = 295,43

Compound **5bba**: Following the **GP** performed with morpholine (69 µL, 0.79 mmol, 1.0 equiv), benzaldehyde (89 µL, 0.87 mmol, 1.1 equiv) and a solution of alkylkzinc bromide in 2-MeTHF (3.5 mL, 0.68 M, 2.38 mmol, 3.0 equiv), the desired product was obtained after purification by FC [V(SiO$_2$) = 25 mL, Cy/EA : 9/1 (100 mL), 8/2 (150 mL)] as a colorless oil (132 mg, 56%).

R*f* (SiO$_2$, Cy/EA : 8/2): 0.31.
HRMS (ESI+): m/z [M+H]$^+$ Calcd. for $C_{20}H_{26}NO$: 296.2009. Found: 296.2005.
^{1}H NMR (400 MHz, CDCl$_3$): δ 7.44 – 7.40 (m, 2H), 7.37 – 7.24 (m, 6H), 7.20 (d, *J* = 8.0 Hz, 2H), 3.77 (t, *J* = 4.6 Hz, 4H), 3.34 (dd, *J* = 9.2, 4.9 Hz, 1H), 2.66 (dt, *J* = 14.6, 7.3 Hz, 2H), 2.54 – 2.43 (m, 4H), 2.10 – 1.97 (m, 1H), 1.89 – 1.80 (m, 1H), 1.67 – 1.47 (m, 2H).
^{13}C{^{1}H} NMR (100 MHz, CDCl$_3$): δ 142.3 (C), 140.4 (C), 128.6 (3 CH), 128.4 (2 CH), 128.3 (CH), 128.1 (2 CH), 127.2 (CH), 125.78 (CH), 70.5 (CH), 67.3 (2 CH$_2$), 51.1 (2 CH$_2$), 35.9 (CH$_2$), 32.0 (CH$_2$), 27.9 (CH$_2$).

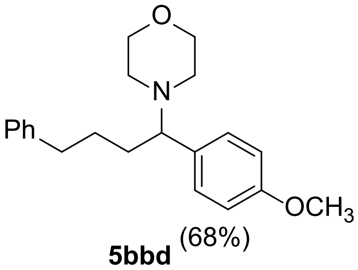

5bbd (68%)
$C_{21}H_{27}NO_2$
MW = 325,45

Compound **5bbd**: Following the **GP** performed with morpholine (69 µL, 0.79 mmol, 1.0 equiv), *p*-anisaldehyde (106 µL, 0.87 mmol, 1.1 equiv) and a solution of alkylkzinc bromide in 2-MeTHF (3.5 mL, 0.68 M, 2.38 mmol, 3.0 equiv), the desired product was obtained after purification by FC [V(SiO_2) = 25 mL, Cy/EA : 8/2 (100 mL), 6/4 (150 mL)] as a pale yellow oil (175 mg, 68%).

R_f (SiO_2, Cy/EA : 8/2): 0.15.
HRMS (ESI+): m/z $[M+H]^+$ Calcd. for $C_{21}H_{28}NO_2$: 326.2115. Found: 326.2117.
^{1}H NMR (400 MHz, $CDCl_3$): δ 7.40 – 7.25 (m, 4H), 7.24 – 7.19 (m, 3H), 6.96 (d, *J* = 8.6 Hz, 2H), 3.88 (s, 3H), 3.77 – 3.71 (m, 4H), 3.29 (dd, *J* = 9.4, 4.7 Hz, 1H), 2.82 – 2.78 (m, 1H), 2.62 – 2.60 (m, 2H), 2.52 – 2.41 (m, 3H), 2.08 – 1.94 (m, 2H), 1.87 – 1.77 (m, 1H), 1.65 – 1.46 (m, 1H).
^{13}C{^{1}H} NMR (100 MHz, $CDCl_3$): δ 158.6 (C), 146.3 (C), 132.2 (C), 129.5 (2 CH), 128.3 (2 CH), 128.2 (2 CH), 125.6 (CH), 113.4 (2 CH), 69.7 (CH), 67.1 (2 CH_2), 55.1 (CH_3), 51.0 (2 CH_2), 35.8 (CH_2), 31.9 (CH_2), 18.0 (CH_2).

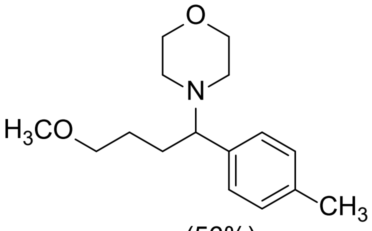

5cbb (56%)
$C_{16}H_{25}NO_2$
MW = 263,38

Compound **5cbb**: Following the **GP** performed with morpholine (78 µL, 0.89 mmol, 1.0 equiv), *p*-tolualdehyde (115 µL, 0.97 mmol, 1.1 equiv) and a solution of alkylkzinc bromide in THF (3.5 mL, 0.76 M, 2.66 mmol, 3.0 equiv), the desired product was obtained after purification by FC [V(SiO_2) = 25 mL, Cy/EA : 8/2 (100 mL), 6/4 (150 mL)] as a pale yellow oil (132 mg, 56%).

R_f (SiO_2, Cy/EA : 6/4): 0.24.
HRMS (ESI+): m/z $[M+H]^+$ Calcd. for $C_{16}H_{26}NO_2$: 264.1958. Found: 264.1957.
^{1}H NMR (400 MHz, $CDCl_3$): δ 7.15 (br s, 4H), 3.70 (t, *J* = 4.5 Hz, 4H), 3.33 (t, *J* = 6.5 Hz, 2H), 3.30 (s, 3H), 3.24 (dd, *J* = 9.2, 4.9 Hz, 1H), 2.50 – 2.40 (m, 4H), 2.36 (s, 3H), 2.07 – 1.96 (m, 1H), 1.81 – 1.71 (m, 1H), 1.52 – 1.34 (m, 2H).
^{13}C{^{1}H} NMR (100 MHz, $CDCl_3$): δ 137.1 (C), 136.6 (C), 128.8 (2 CH), 128.5 (2 CH), 77.7 (CH_2), 70.0 (CH), 67.2 (2 CH_2), 58.4 (CH_3), 51.0 (2 CH_2), 29.0 (CH_2), 26.4 (CH_2), 21.0 (CH_3).

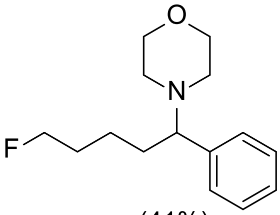

5eba (41%)
$C_{15}H_{22}FNO$
MW = 251,35

Compound **5eba**: Following the **GP** performed with morpholine (62 µL, 0.71 mmol, 1.0 equiv), benzaldehyde (80 µL, 0.78 mmol, 1.1 equiv) and a solution of alkylkzinc bromide in 2-MeTHF (3.5 mL, 0.61 M, 2.13 mmol, 3.0 equiv), the desired product was obtained after purification by FC [V(SiO_2) = 25 mL, Cy/EA : 8/2 (150 mL), 6/4 (100 mL)] as a pale yellow oil (74 mg, 41%).

R_f (SiO_2, Cy/EA : 8/2): 0.20.
HRMS (ESI+): m/z $[M+H]^+$ Calcd. for $C_{15}H_{23}FNO$: 252.1758. Found: 252.1761.
^{1}H NMR (400 MHz, $CDCl_3$): δ 7.39 – 7.35 (m, 2H), 7.32 – 7.27 (m, 3H), 4.46 (t, *J* = 6.2 Hz, 1H), 4.35 (t, *J* = 6.2 Hz, 1H), 3.73 (t, *J* = 4.7 Hz, 4H), 3.29 (dd, *J* = 9.2, 4.7 Hz, 1H), 2.52 – 2.40 (m, 4H), 2.04 – 1.96 (m, 1H), 1.85 – 1.63 (m, 3H), 1.38 – 1.17 (m, 2H).
^{13}C{^{1}H} NMR (100 MHz, $CDCl_3$): δ 140.2 (C), 128.7 (2 CH), 128.2 (2 CH), 127.3 (CH), 84.0 (d, $^1J_{C-F}$ = 164.2 Hz, CH_2), 70.5 (CH), 67.2 (2 CH_2), 51.1 (2 CH_2), 32.1 (CH_2), 30.5 (d, $^2J_{C-F}$ = 18.6 Hz, CH_2), 22.0 (d, $^3J_{C-F}$ = 4.8 Hz, CH_2).
^{19}F{^{1}H} NMR (376 MHz, $CDCl_3$): δ –112.1 (F).

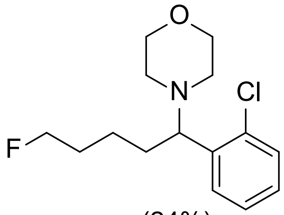

5ebe (24%)
$C_{15}H_{21}ClFNO$
MW = 285,79

Compound **5ebe**: Following the **GP** performed with morpholine (62 µL, 0.71 mmol, 1.0 equiv), 2-chlorobenzaldehyde (88 µL, 0.78 mmol, 1.1 equiv) and a solution of alkylkzinc bromide in 2-MeTHF (3.5 mL, 0.61 M, 2.13 mmol, 3.0 equiv), the desired product was obtained after purification by FC [V(SiO_2) = 25 mL, Cy/EA : 8/2 (150 mL), 6/4 (100 mL)] as a colorless oil (48 mg, 24%).

R_f (SiO_2, Cy/EA : 8/2): 0.15.
HRMS (ESI+): m/z $[M+H]^+$ Calcd. for $C_{15}H_{22}ClFNO$: 286.1368. Found: 286.1369.
^{1}H NMR (400 MHz, $CDCl_3$): δ 7.43 (dd, J = 7.9, 1.8 Hz, 1H), 7.35 (dd, J = 7.9, 1.4 Hz, 1H), 7.25 (td, J = 7.6, 1.4 Hz, 1H), 7.16 (td, J = 7.6, 1.8 Hz, 1H), 4.41 (t, J = 6.1 Hz, 1H), 4.30 (t, J = 6.1 Hz, 1H), 3.95 (dd, J = 8.9, 4.6 Hz, 1H), 3.66 (t, J = 4.6 Hz, 4H), 2.55 – 2.52 (m, 2H), 2.37 (dt, J = 11.0, 4.7 Hz, 2H), 1.97 – 1.88 (m, 1H), 1.76 – 1.56 (m, 3H), 1.33 – 1.14 (m, 2H).
^{13}C{^{1}H} NMR (100 MHz, $CDCl_3$): δ 138.6 (C), 135.0 (C), 129.6 (CH), 129.2 (CH), 128.1 (CH), 126.8 (CH), 84.0 (d, $^1J_{C-F}$ = 164.3 Hz, CH_2), 67.3 (2 CH_2), 64.5 (CH), 51.1 (2 CH_2), 31.8 (CH_2), 30.6 (d, $^2J_{C-F}$ = 19.8 Hz, CH_2), 21.1 (d, $^3J_{C-F}$ = 4.8 Hz, CH_2).
^{19}F{^{1}H} NMR (376 MHz, $CDCl_3$): δ –112.1 (F).

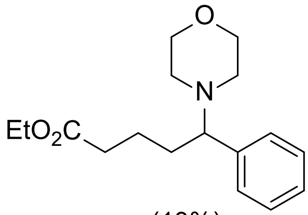

5fba (13%)
$C_{17}H_{25}NO_3$
MW = 291,39

Compound **5fba**:[2] Following the **GP** performed with morpholine (76 µL, 0.86 mmol, 1.0 equiv), benzaldehyde (97 µL, 0.95 mmol, 1.1 equiv) and a solution of alkylkzinc bromide in 2-MeTHF (7.0 mL, 0.37 M, 2.59 mmol, 3.0 equiv), the desired product was obtained after purification by FC [V(SiO_2) = 25 mL, Cy/EA : 8/2 (100 mL), 6/4 (150 mL)] as a pale yellow oil (33 mg, 13%).

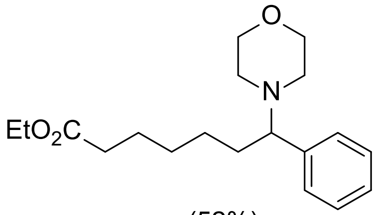

5gba (52%)
$C_{19}H_{29}NO_3$
MW = 319,45

Compound **5gba**: Following the **GP** performed with morpholine (67 µL, 0.77 mmol, 1.0 equiv), benzaldehyde (86 µL, 0.85 mmol, 1.1 equiv) and a solution of alkylkzinc bromide in 2-MeTHF (3.5 mL, 0.66 M, 2.31 mmol, 3.0 equiv), the desired product was obtained after purification by FC [V(SiO_2) = 25 mL, Cy/EA : 8/2 (100 mL), 6/4 (150 mL)] as a pale yellow oil (128 mg, 52%).

R_f (SiO_2, Cy/EA : 6/4): 0.40.
HRMS (ESI+): m/z $[M+H]^+$ Calcd. for $C_{19}H_{30}NO_3$: 320.2220. Found: 320.2223.
^{1}H NMR (400 MHz, $CDCl_3$): δ 7.36 – 7.32 (m, 2H), 7.30 – 7.24 (m, 3H), 4.12 (q, J = 7.1 Hz, 2H), 3.71 (t, J = 4.6 Hz, 4H), 3.26 (dd, J = 9.5, 4.6 Hz, 1H), 2.51 – 2.38 (m, 4H), 2.24 (t, J = 7.5 Hz, 2H), 1.98 – 1.89 (m, 1H), 1.79 – 1.67 (m, 1H), 1.61 – 1.53 (m, 2H), 1.44 – 1.08 (m, 7H).
^{13}C{^{1}H} NMR (100 MHz, $CDCl_3$): δ 173.8 (C), 140.2 (C), 128.7 (2 CH), 128.2 (2 CH), 127.2 (CH), 70.6 (CH), 67.1 (2 CH_2), 60.2 (CH_2), 51.1 (2 CH_2), 34.3 (CH_2), 32.1 (CH_2), 29.2 (CH_2), 25.9 (CH_2), 24.8 (CH_2), 14.3 (CH_3).

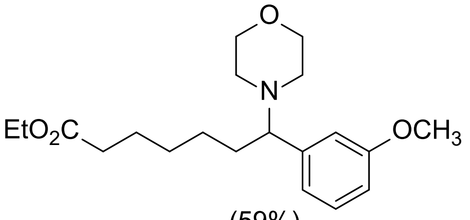

(59%)
5gbf
$C_{20}H_{31}NO_4$
MW = 349,47

Compound **5gbf**: Following the **GP** performed with morpholine (67 µL, 0.77 mmol, 1.0 equiv), 3-anisaldehyde (103 µL, 0.85 mmol, 1.1 equiv) and a solution of alkylkzinc bromide in 2-MeTHF (3.5 mL, 0.66 M, 2.31 mmol, 3.0 equiv), the desired product was obtained after purification by FC [V(SiO_2) = 25 mL, Cy/EA : 8/2 (150 mL), 6/4 (100 mL)] as a pale yellow oil (158 mg, 59%).

R_f (SiO_2, Cy/EA : 6/4): 0.32.
HRMS (ESI+): m/z $[M+H]^+$ Calcd. for $C_{20}H_{32}NO_4$: 350.2326. Found: 350.2328.
^{1}H NMR (400 MHz, $CDCl_3$): δ 7.20 – 7.16 (m, 1H), 6.78 – 6.75 (m, 3H), 4.06 (q, *J* = 7.1 Hz, 2H), 3.77 (s, 3H), 3.63 (t, *J* = 4.5 Hz, 4H), 3.13 (dd, *J* = 9.4, 4.6 Hz, 1H), 2.46 – 2.31 (m, 4H), 2.18 (t, *J* = 7.5 Hz, 2H), 1.88 – 1.80 (m, 1H), 1.68 – 1.60 (m, 1H), 1.54 – 1.47 (m, 2H), 1.38 – 1.02 (m, 7H).
^{13}C{^{1}H} NMR (100 MHz, $CDCl_3$): δ 173.7 (C), 159.5 (C), 142.2 (C), 129.0 (CH), 121.1 (CH), 114.4 (CH), 112.1 (CH), 70.6 (CH), 67.2 (2 CH_2), 60.2 (CH_2), 55.2 (CH_3), 51.2 (2 CH_2), 34.3 (CH_2), 32.2 (CH_2), 29.2 (CH_2), 25.9 (CH_2), 24.8 (CH_2), 14.3 (CH_3).

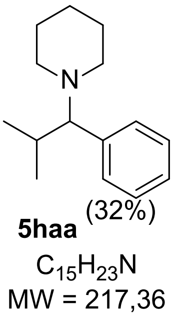

5haa (32%)
$C_{15}H_{23}N$
MW = 217,36

Compound **5haa**:[1] Following the **GP** performed with piperidine (48 µL, 0.48 mmol, 1.0 equiv), benzaldehyde (54 µL, 0.53 mmol, 1.1 equiv) and a solution of alkylkzinc bromide in THF (2.5 mL, 0.58 M, 1.45 mmol, 3.0 equiv), the desired product was obtained after purification by FC [V(SiO_2) = 25 mL, Cy/EA : 9.5/0.5 (150 mL), 9/1 (100 mL)] as a pale yellow oil (33 mg, 32%).

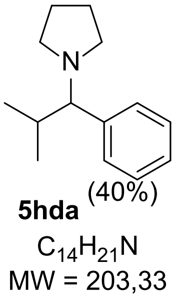

5hda (40%)
$C_{14}H_{21}N$
MW = 203,33

Compound **5hda**:[1] Following the **GP** performed with pyrrolidine (51 µL, 0.61 mmol, 1.0 equiv), benzaldehyde (68 µL, 0.67 mmol, 1.1 equiv) and a solution of alkylkzinc bromide in THF (3.5 mL, 0.52 M, 1.82 mmol, 3.0 equiv), the desired product was obtained after purification by FC [V(SiO_2) = 25 mL, Cy/EA : 9.5/0.5 (100 mL), 9/1 (150 mL)] as a colorless oil (50 mg, 40%).

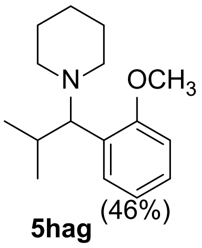

5hag (46%)
$C_{16}H_{25}NO$
MW = 247,38

Compound **5hag**:[1] Following the **GP** performed with piperidine (48 µL, 0.48 mmol, 1.0 equiv), 2-anisaldehyde (64 µL, 0.53 mmol, 1.1 equiv) and a solution of alkylkzinc bromide in THF (2.5 mL, 0.58 M, 1.45 mmol, 3.0 equiv), the desired product was obtained after purification by FC [V(SiO_2) = 25 mL, Cy/EA : 9.5/0.5 (100 mL), 9/1 (150 mL)] as a colorless oil (57 mg, 46%).

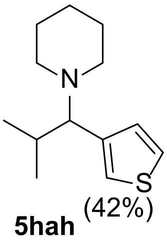

5hah (42%)
$C_{13}H_{21}NS$
MW = 223,38

Compound **5hah**:[1] Following the **GP** performed with piperidine (48 µL, 0.48 mmol, 1.0 equiv), 3-thiophenecarboxaldehyde (47 µL, 0.53 mmol, 1.1 equiv) and a solution of alkylkzinc bromide in THF (2.5 mL, 0.58 M, 1.45 mmol, 3.0 equiv), the desired product was obtained after purification by FC [V(SiO_2) = 25 mL, Cy/EA : 9.5/0.5 (150 mL), 9/1 (100 mL)] as a colorless oil (55 mg, 42%).

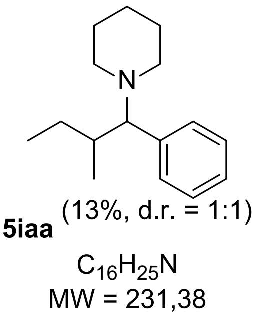

5iaa (13%, d.r. = 1:1)

$C_{16}H_{25}N$
MW = 231,38

Compound **5iaa**:[1] Following the **GP** performed with piperidine (46 µL, 0.46 mmol, 1.0 equiv), benzaldehyde (52 µL, 0.51 mmol, 1.1 equiv) and a solution of alkylkzinc bromide in THF (2.0 mL, 0.69 M, 1.38 mmol, 3.0 equiv), the desired product was obtained after purification by FC [V(SiO_2) = 25 mL, Cy/EA : 9.5/0.5 (150 mL), 9/1 (100 mL)] as a colorless oil (14 mg, 13%, d.r. = 1:1).

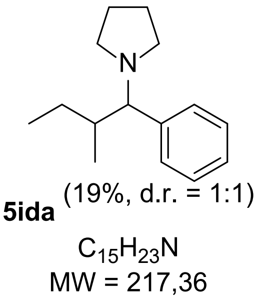

5ida (19%, d.r. = 1:1)

$C_{15}H_{23}N$
MW = 217,36

Compound **5ida**: Following the **GP** performed with pyrrolidine (39 µL, 0.46 mmol, 1.0 equiv), benzaldehyde (52 µL, 0.51 mmol, 1.1 equiv) and a solution of alkylkzinc bromide in THF (2.0 mL, 0.69 M, 1.38 mmol, 3.0 equiv), the desired product was obtained after purification by FC [V(SiO_2) = 25 mL, Cy/EA : 9.5/0.5 (150 mL), 9/1 (100 mL)] as a colorless oil (19 mg, 19%, d.r. = 1:1).

R_f (SiO_2, Cy/EA : 9/1): 0.13.
HRMS (ESI+): m/z $[M+H]^+$ Calcd. for $C_{15}H_{24}N$: 218.1903. Found: 218.1904.
^{1}H NMR (400 MHz, $CDCl_3$): δ 7.34 – 7.28 (m, 10H), 3.20 (d, *J* = 4.8 Hz, 1H), 3.14 (d, *J* = 5.4 Hz, 1H), 2.56 – 2.48 (m, 8H), 2.03 – 1.85 (m, 4H), 1.82 – 1.76 (m, 8H), 1.43 – 1.31 (m, 2H), 0.97 – 0.84 (m, 12H).
^{13}C{^{1}H} NMR (100 MHz, $CDCl_3$): δ 139.9 (2 C), 129.5 (2 CH), 129.5 (2 CH), 127.5 (4 CH), 126.8 (CH), 126.8 (CH), 75.7 (CH), 74.8 (CH), 52.6 (2 CH_2), 52.1 (2 CH_2), 37.8 (2 CH), 27.6 (CH_2), 23.3 (2 CH_2), 23.3 (2 CH_2), 23.2 (CH_2), 17.1 (CH_3), 13.5 (CH_3), 12.3 (CH_3), 12.1 (CH_3).

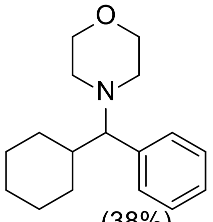

5jba (38%)

$C_{17}H_{25}NO$
MW = 259,39

Compound **5jba**: Following the **GP** performed with morpholine (57 µL, 0.65 mmol, 1.0 equiv), benzaldehyde (73 µL, 0.72 mmol, 1.1 equiv) and a solution of alkylkzinc bromide in 2-MeTHF (3.5 mL, 0.56 M, 1.96 mmol, 3.0 equiv), the desired product was obtained after purification by FC [V(SiO_2) = 25 mL, Cy/EA : 9/0 (150 mL), 8/2 (100 mL)] as a pale yellow oil (65 mg, 38%).

R_f (SiO_2, Cy/EA : 8/2): 0.64.
HRMS (ESI+): m/z $[M+H]^+$ Calcd. for $C_{17}H_{26}NO$: 260.2009. Found: 260.2011.
^{1}H NMR (400 MHz, $CDCl_3$): δ 7.38 – 7.30 (m, 3H), 7.20 (d, *J* = 6.8 Hz, 2H), 3.73 (t, *J* = 5.7 Hz, 4H), 3.14 (d, *J* = 8.1 Hz, 1H), 2.45 – 2.37 (m, 4H), 2.03 – 1.93 (m, 2H), 1.70 (td, *J* = 44.7, 42.2, 13.3 Hz, 4H), 1.37 – 1.11 (m, 3H), 0.87 (dq, *J* = 25.6, 12.8 Hz, 2H).
^{13}C{^{1}H} NMR (100 MHz, $CDCl_3$): δ 137.9 (C), 129.4 (2 CH), 127.7 (2 CH), 126.9 (CH), 75.6 (CH), 67.5 (2 CH_2), 50.6 (2 CH_2), 37.7 (CH), 31.3 (CH_2), 29.1 (CH_2), 26.9 (CH_2), 26.6 (CH_2), 26.4 (CH_2).

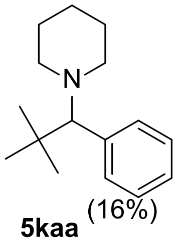

5kaa (16%)

$C_{16}H_{25}N$
MW = 231,38

Compound **5kaa**:[1] Following the **GP** performed with piperidine (32 µL, 0.33 mmol, 1.0 equiv), benzaldehyde (37 µL, 0.36 mmol, 1.1 equiv) and a solution of alkylkzinc bromide in THF (7.0 mL, 0.14 M, 0.98 mmol, 3.0 equiv), the desired product was obtained after purification by FC [V(SiO_2) = 25 mL, Cy/EA : 9.5/0.5 (100 mL), 9/1 (150 mL)] as a pale yellow oil (12 mg, 16%).

6. Copies of NMR spectra

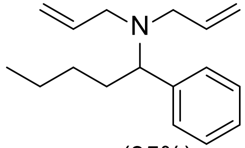

5aca (35%)

^1H NMR (400 MHz / $CDCl_3$)

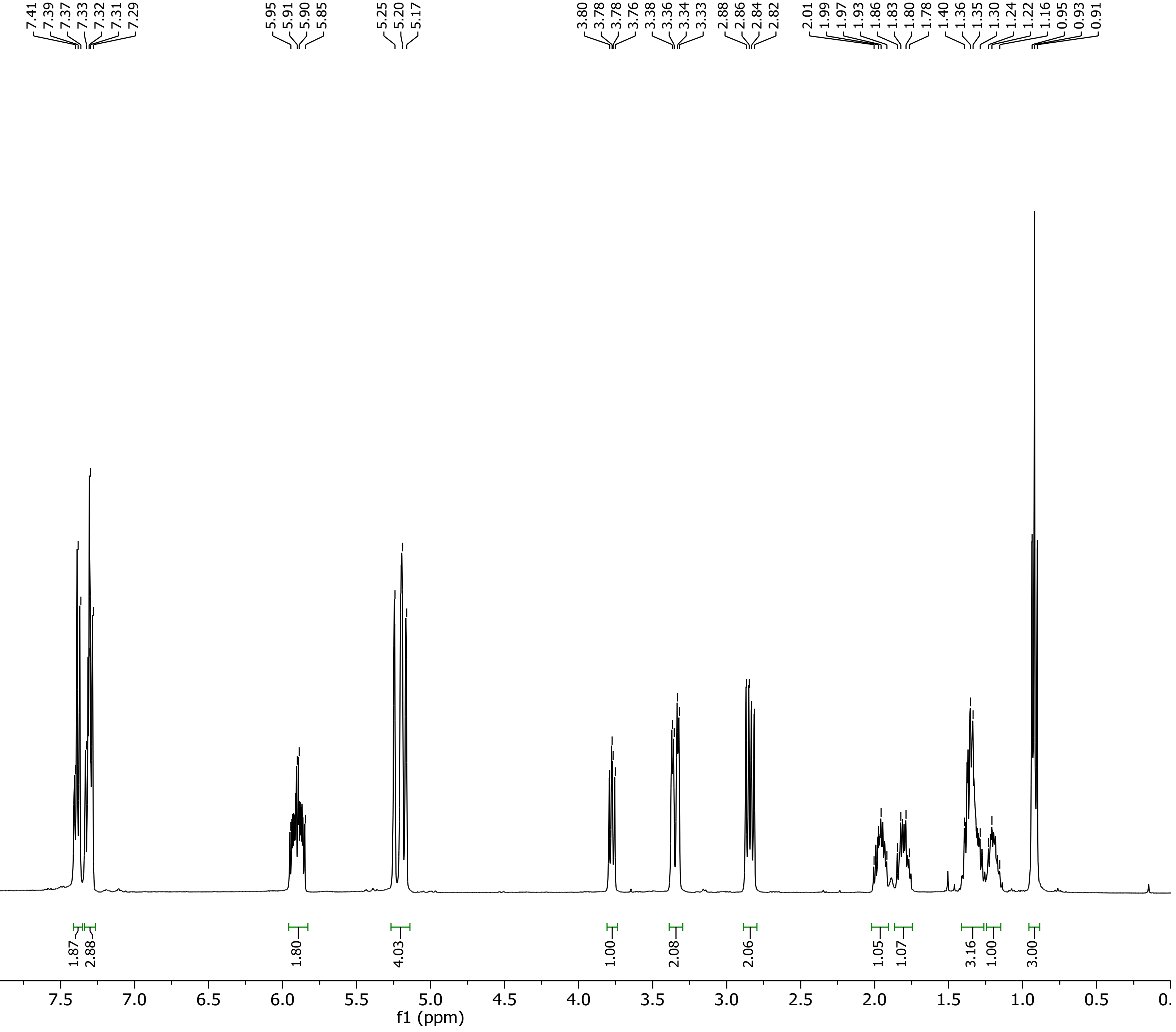

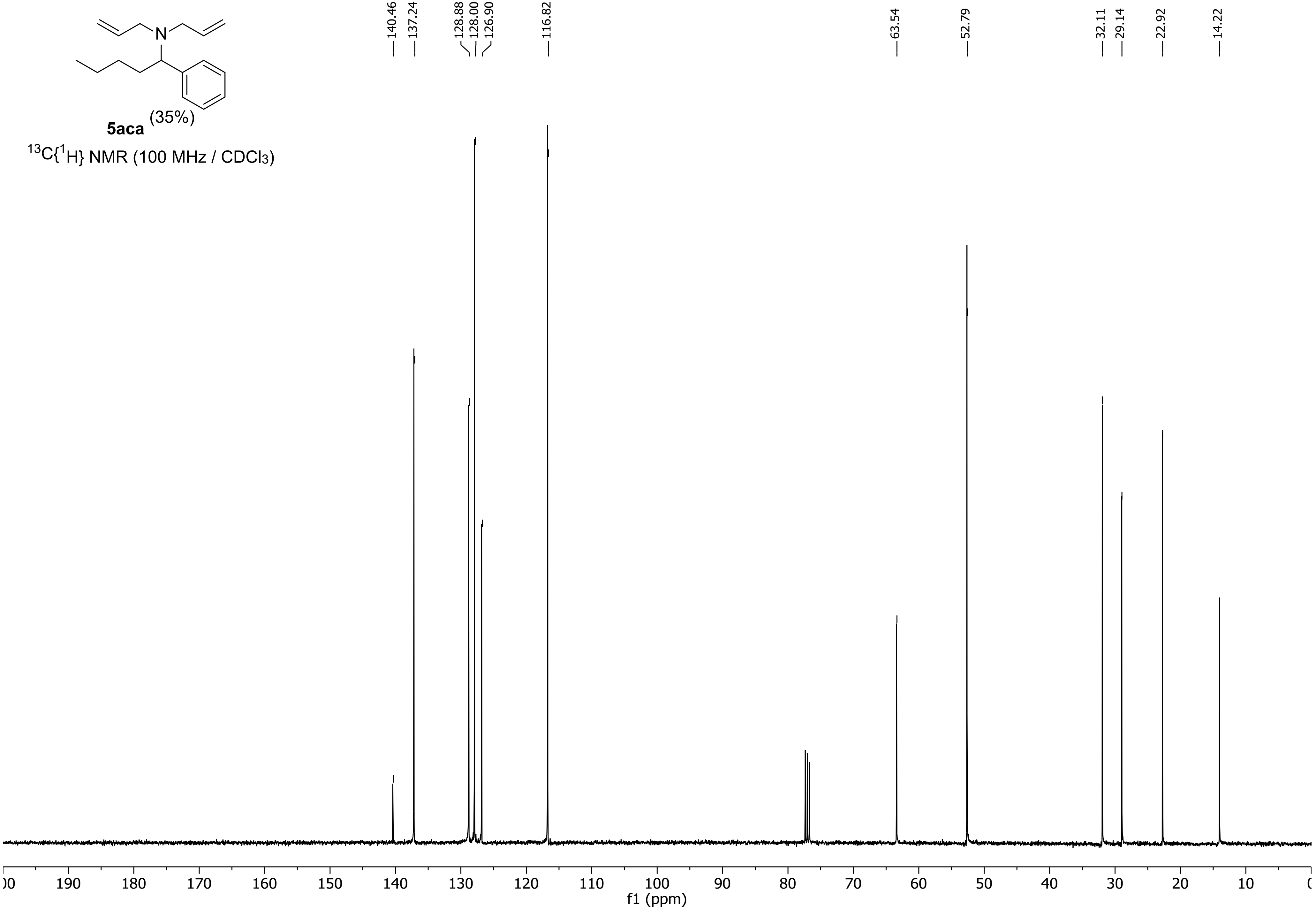

5aca (35%)
$^{13}C\{^1H\}$ NMR (100 MHz / CDCl3)
140.46
137.24
128.88
128.00
126.90
116.82
63.54
52.79
32.11
29.14
22.92
14.22
190
180
170
160
150
140
130
120
110
100
90
80
70
60
50
40
30
20
10
f1 (ppm)

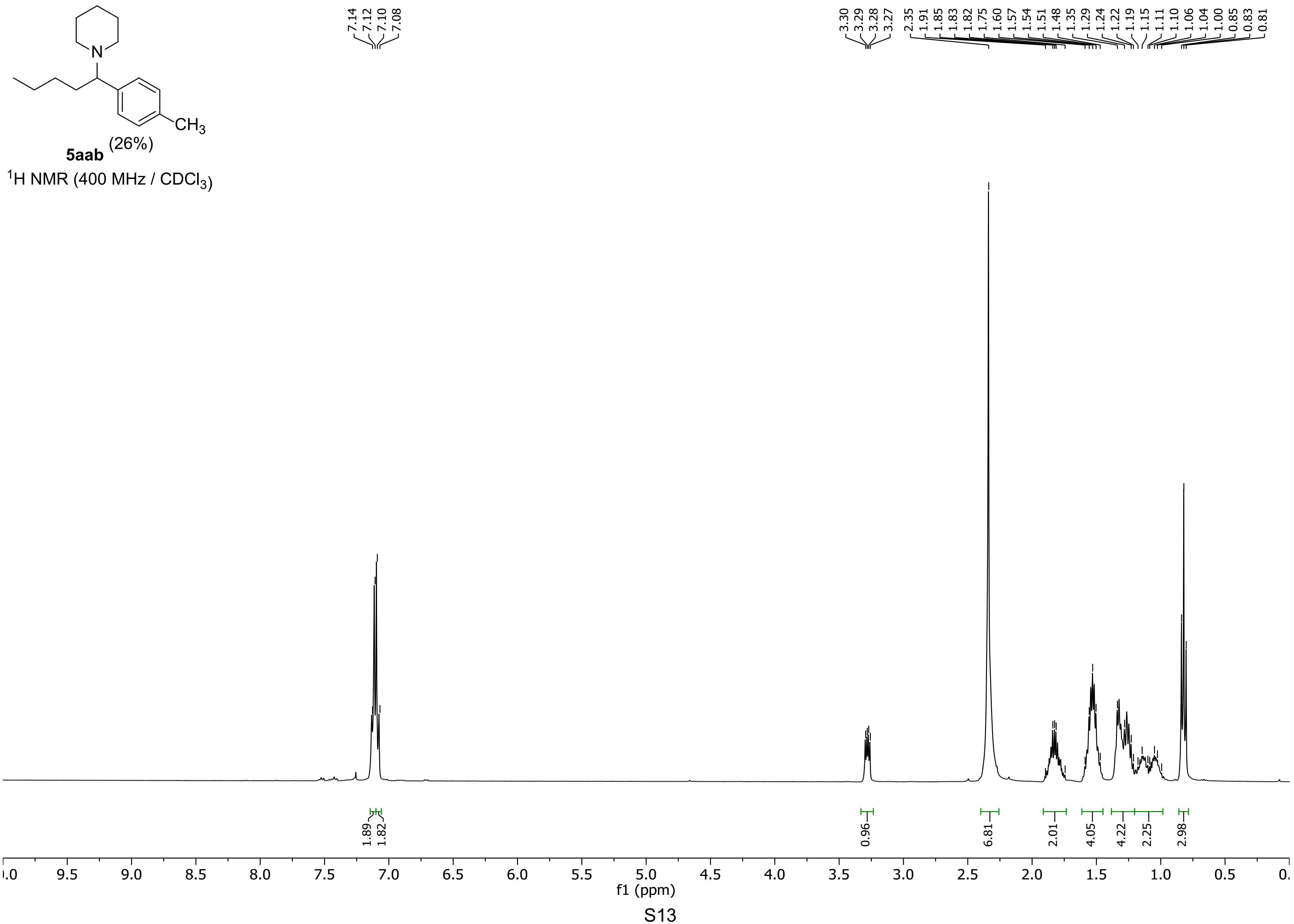

5aab (26%)

^{1}H NMR (400 MHz / $CDCl_3$)

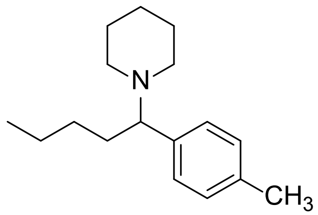

5aab (26%)

$^{13}C\{^1H\}$ NMR (100 MHz / $CDCl_3$)

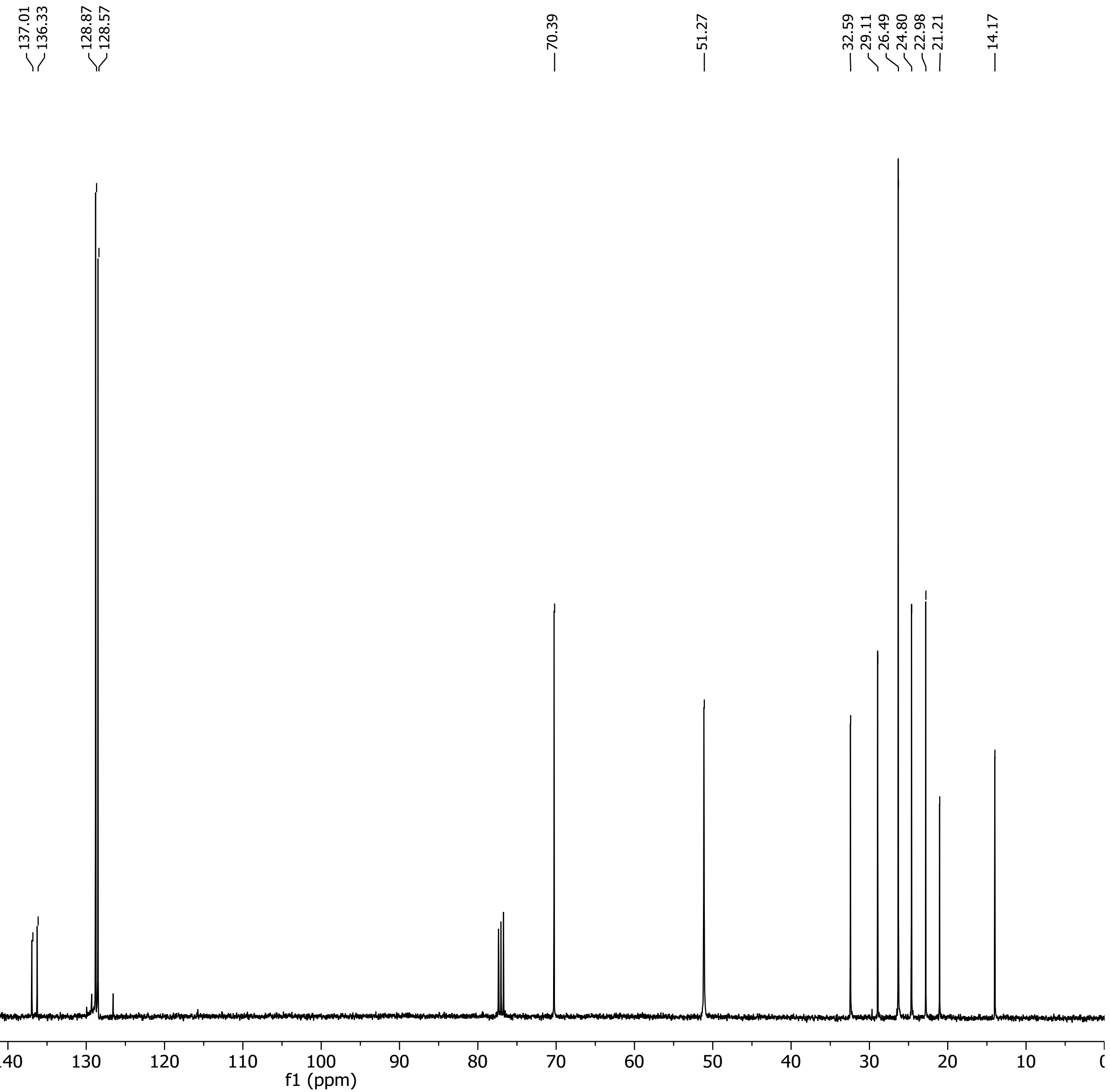

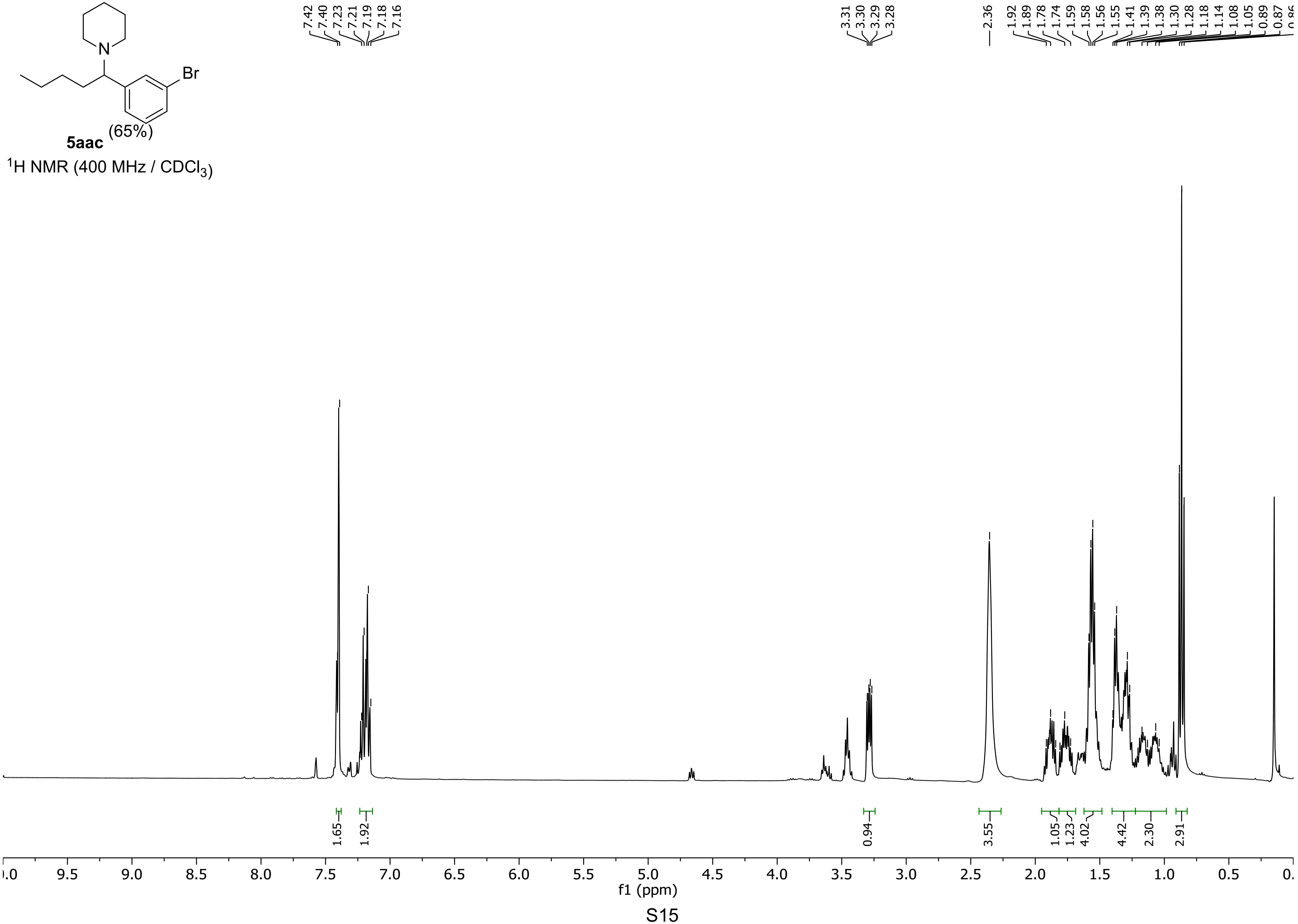
Br
N
5aac (65%)
1H NMR (400 MHz / CDCl3)
7.42
7.40
7.23
7.21
7.19
7.18
7.16
3.31
3.30
3.29
3.28
2.36
1.92
1.89
1.78
1.74
1.59
1.58
1.56
1.55
1.41
1.39
1.38
1.30
1.28
1.18
1.14
1.08
1.05
0.89
0.87
1.65
1.92
0.94
3.55
1.05
1.23
4.02
4.42
2.30
2.91
9.5
9.0
8.5
8.0
7.5
7.0
6.5
6.0
5.5
5.0
4.5
4.0
3.5
3.0
2.5
2.0
1.5
1.0
0.5
f1 (ppm)

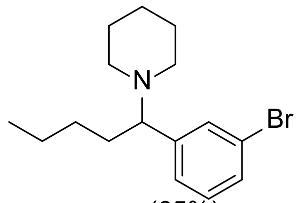

5aac (65%)

$^{13}C\{^1H\}$ NMR (100 MHz / $CDCl_3$)

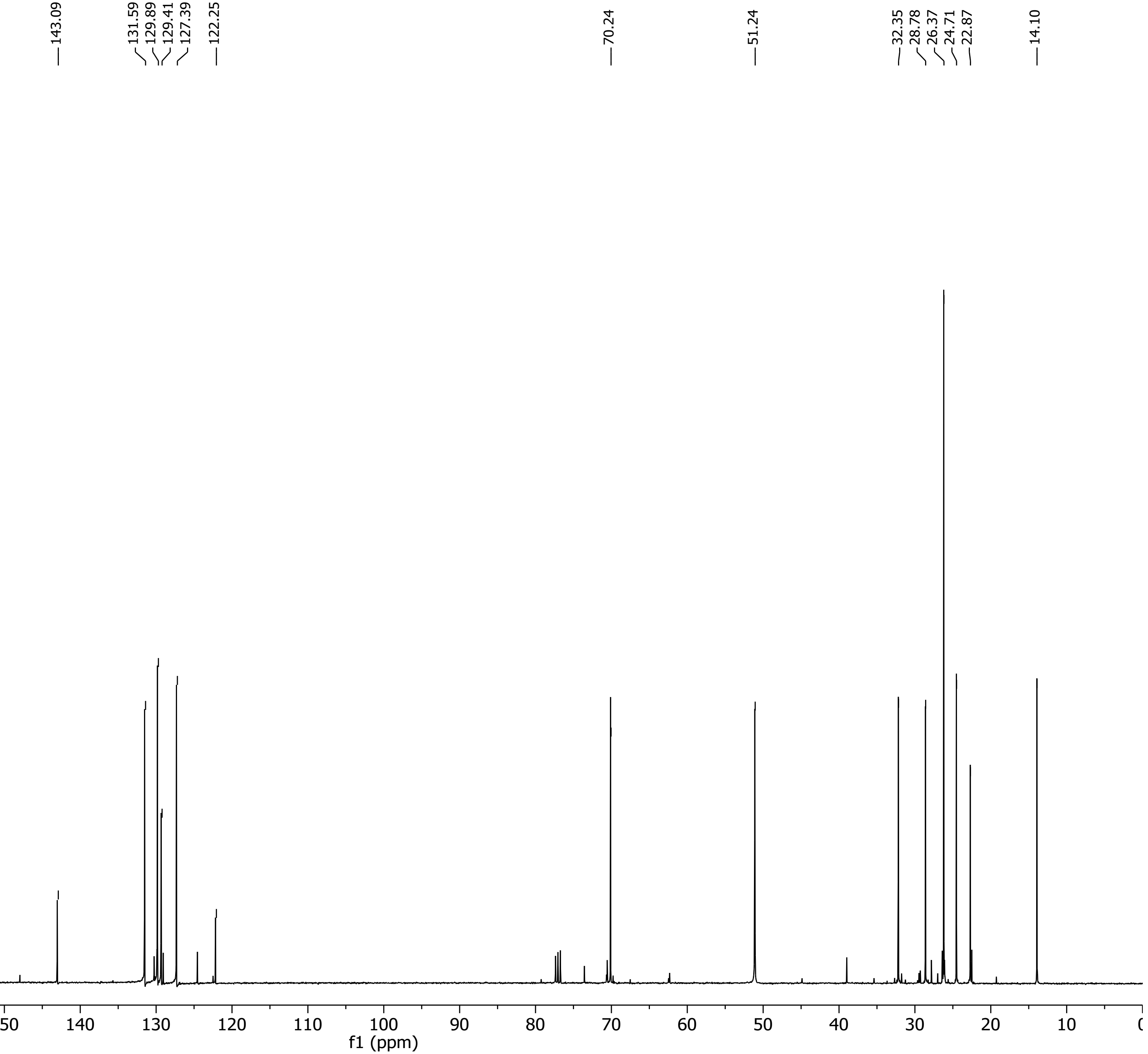

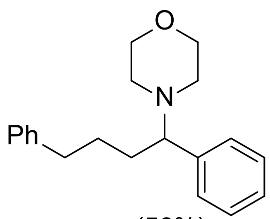

5bba (56%)

^{1}H NMR (400 MHz / $CDCl_3$)

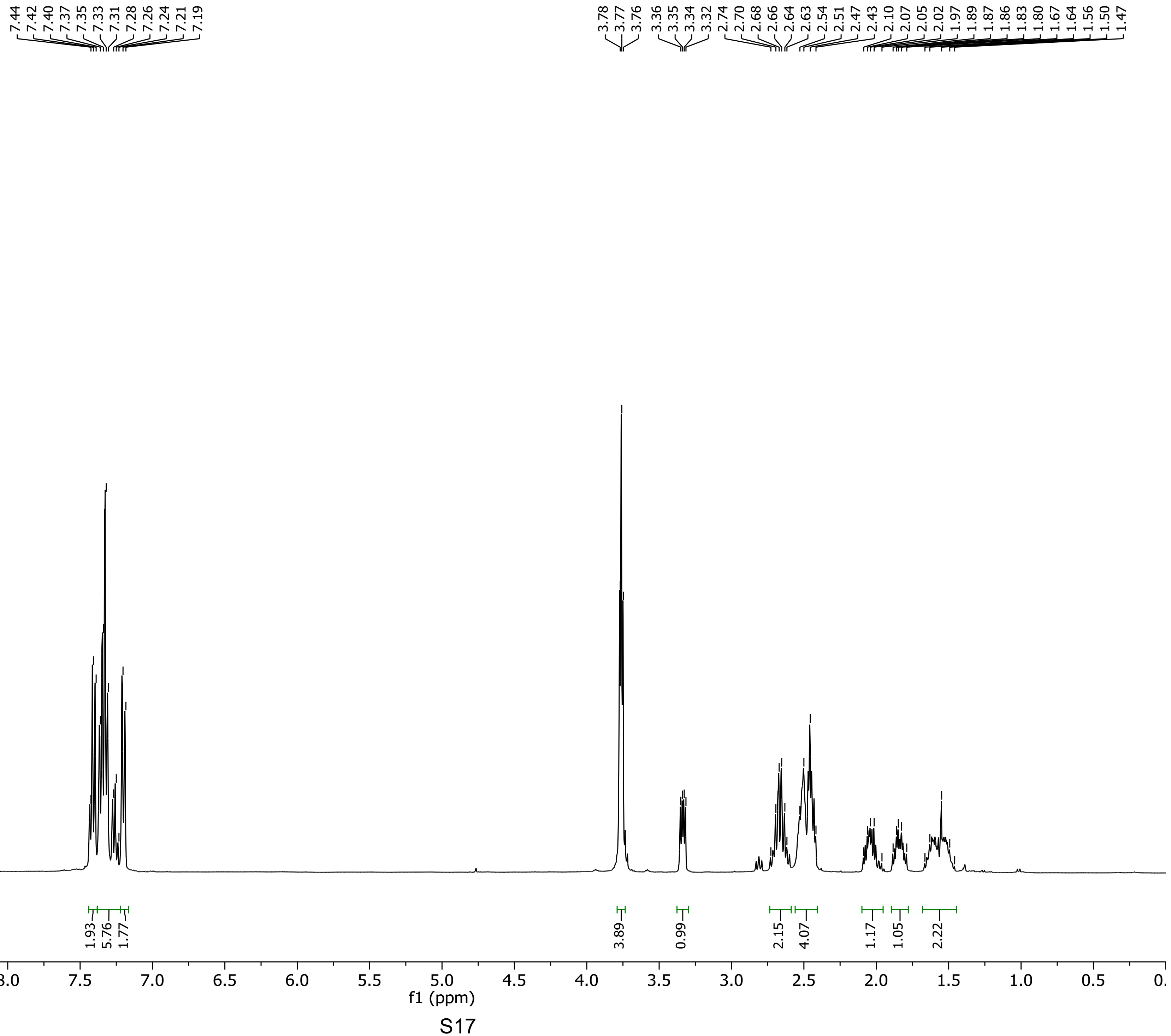

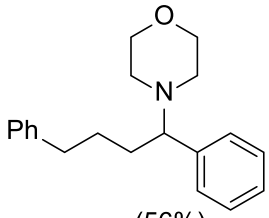

5bba (56%)

$^{13}C\{^1H\}$ NMR (100 MHz / $CDCl_3$)

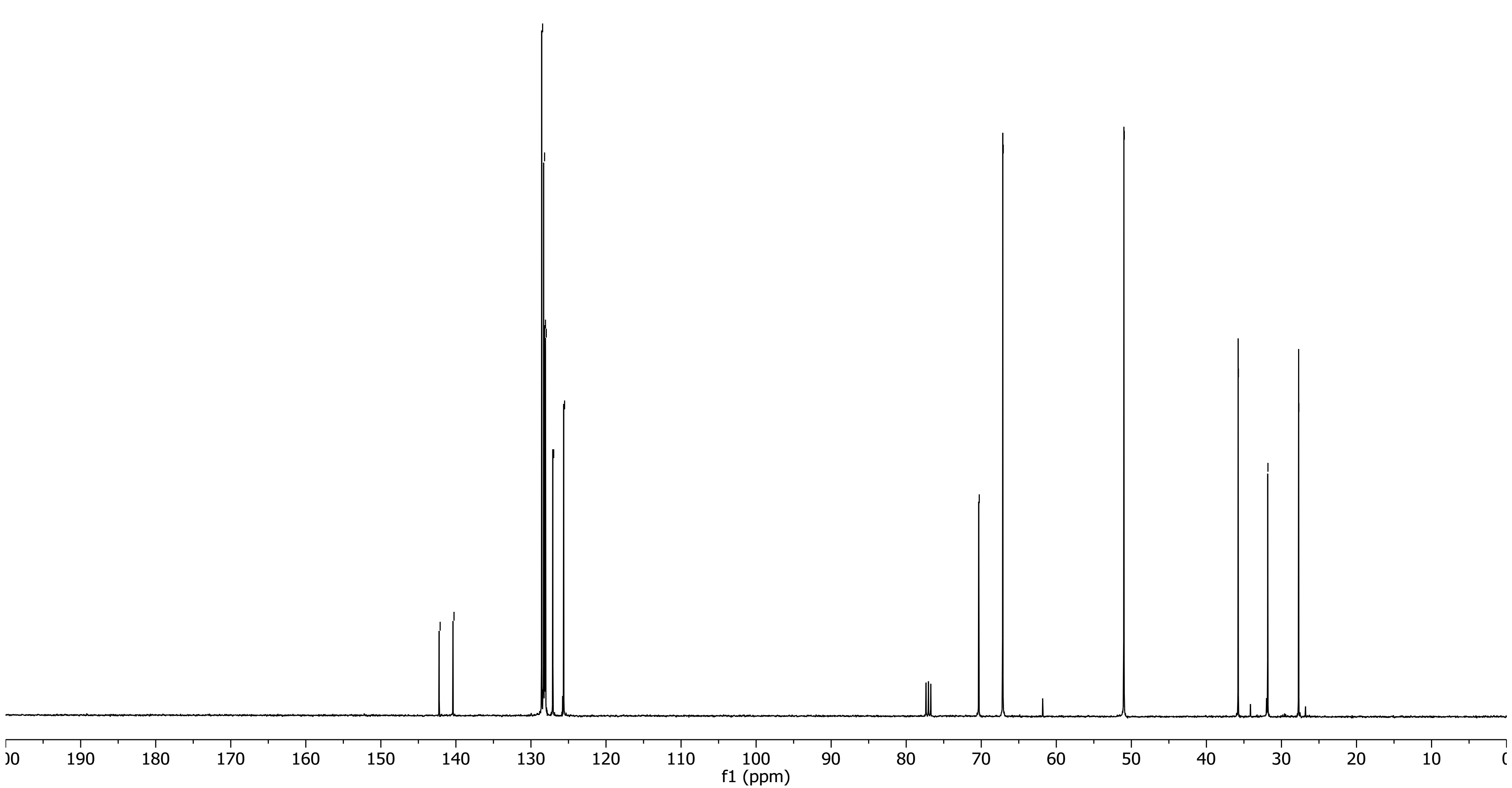

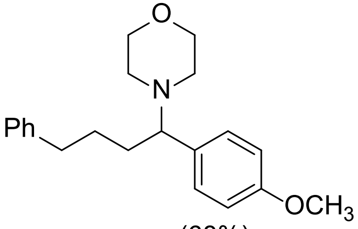

5bbd (68%)

^{1}H NMR (400 MHz / $CDCl_3$)

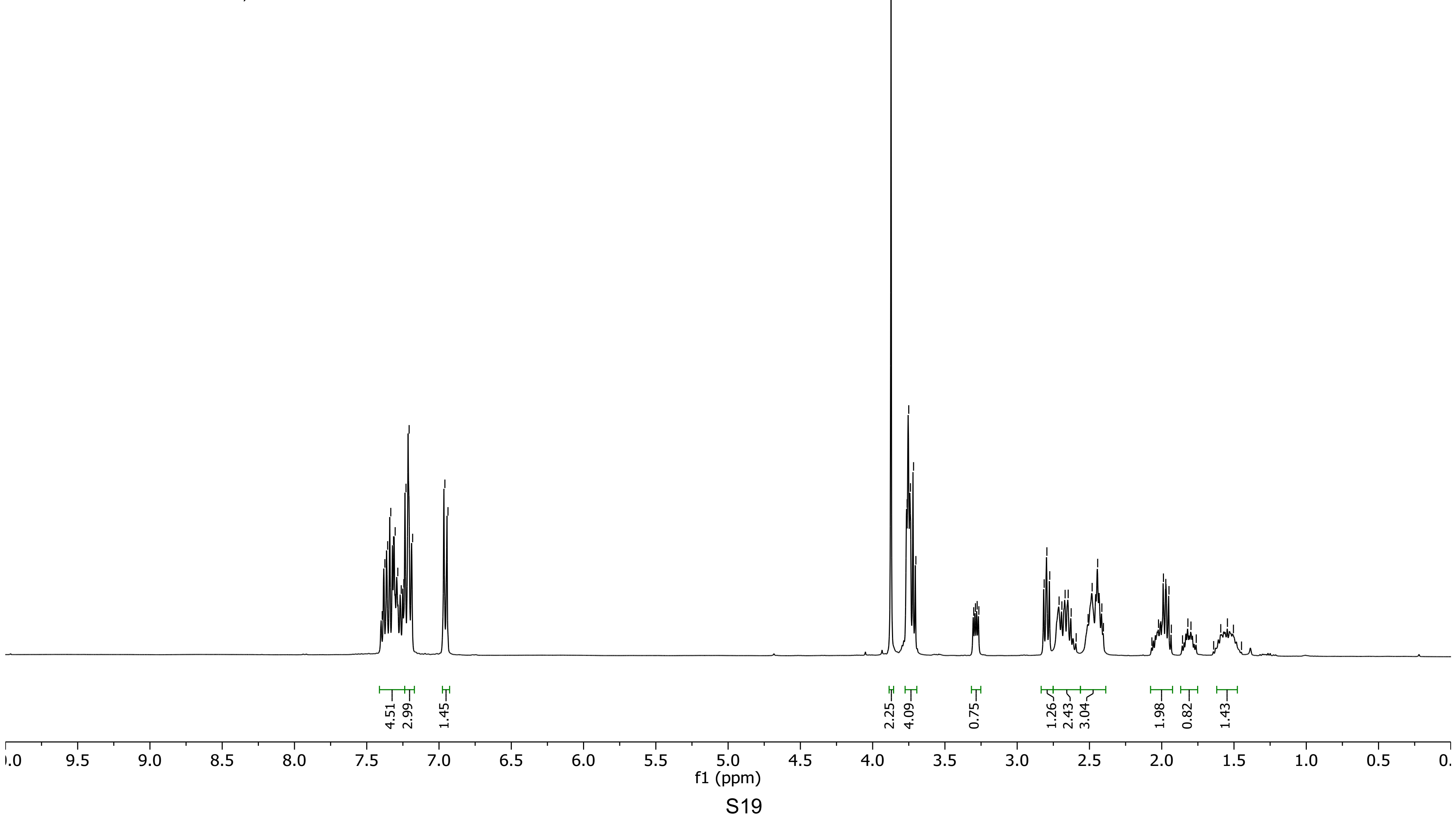

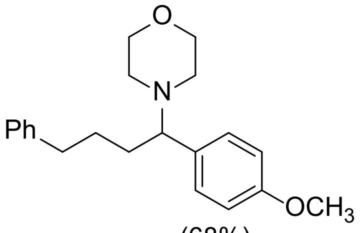

5bbd (68%)

$^{13}C\{^1H\}$ NMR (100 MHz / $CDCl_3$)

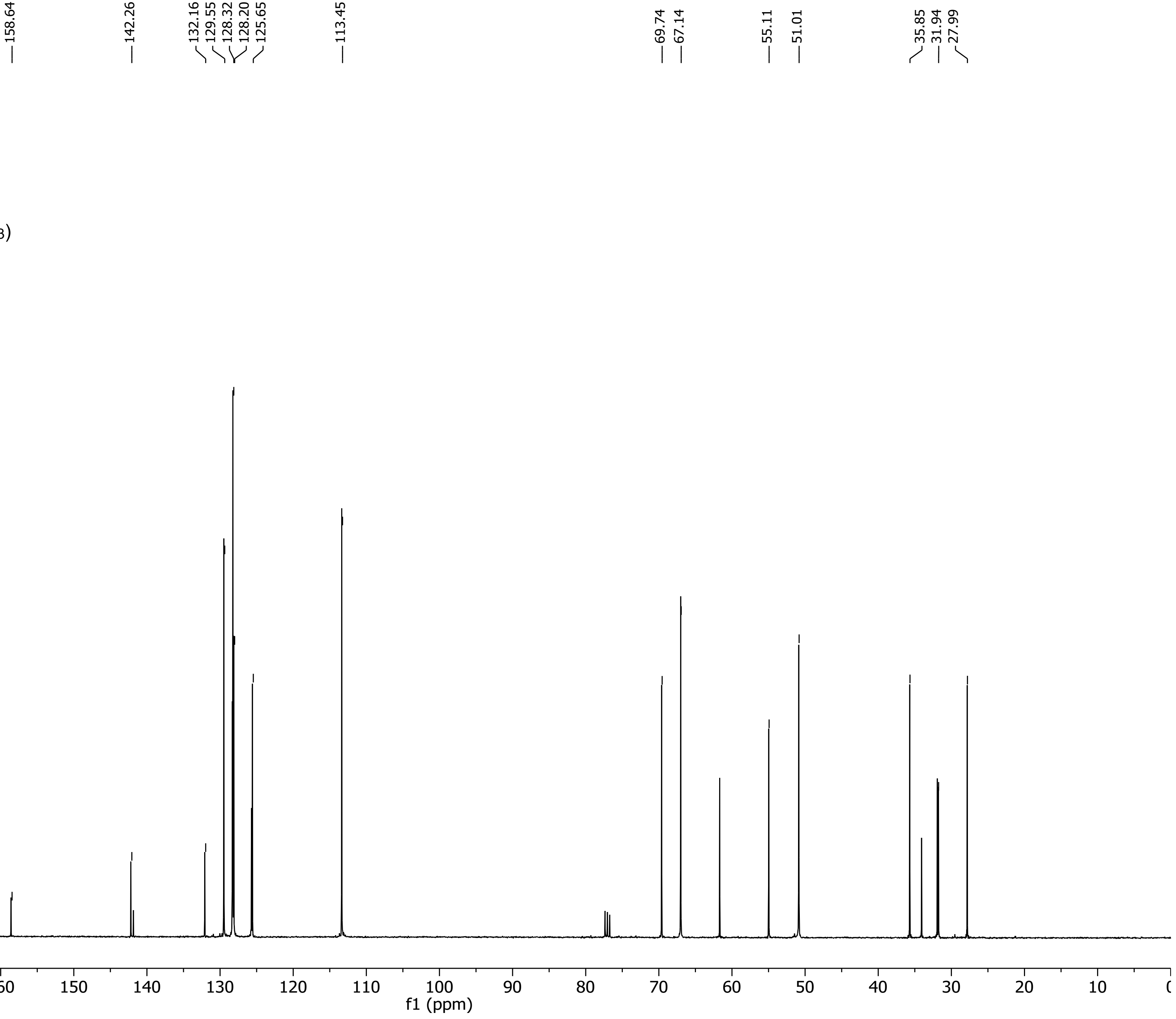

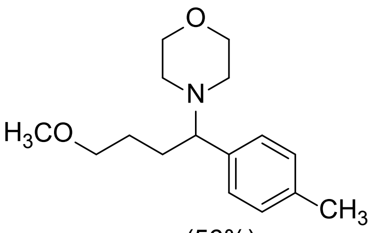

5cbb (56%)

^1H NMR (400 MHz / $CDCl_3$)

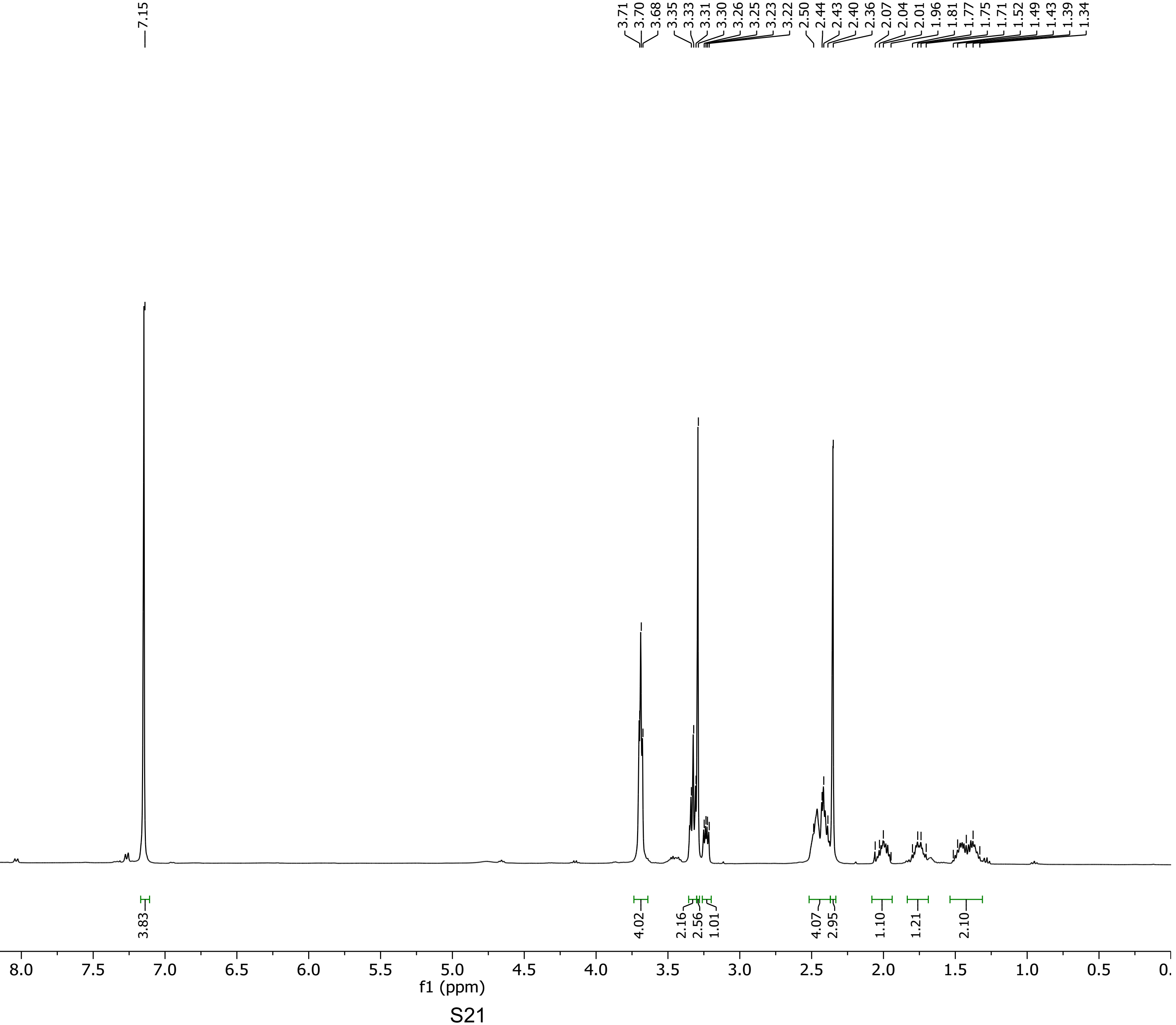

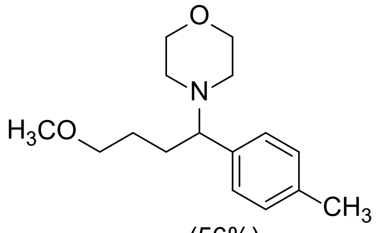

5cbb (56%)

$^{13}C\{^1H\}$ NMR (100 MHz / $CDCl_3$)

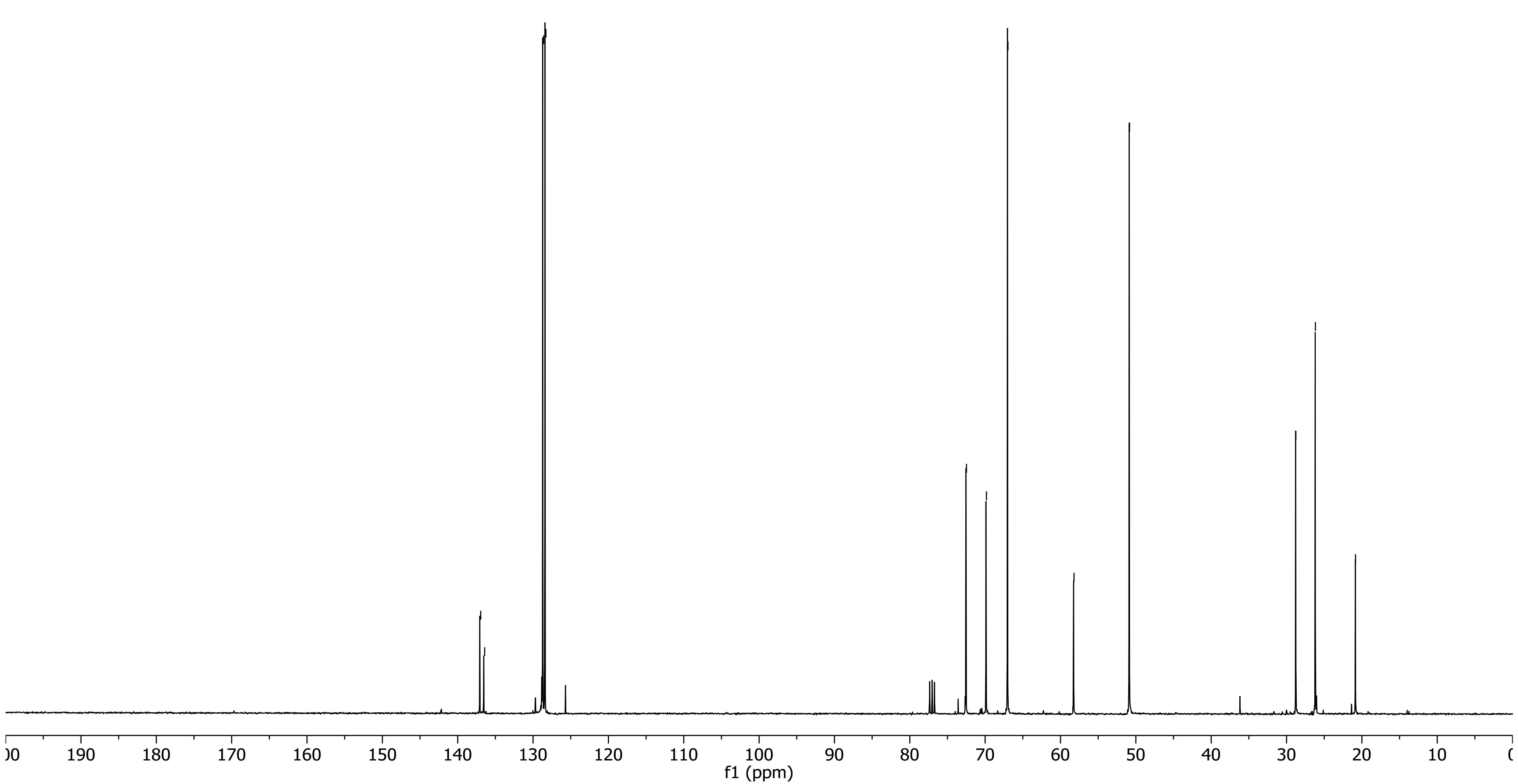

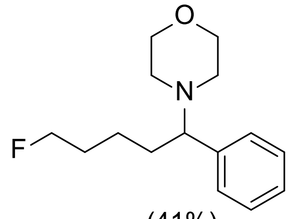

5eba (41%)

^{1}H NMR (400 MHz / $CDCl_3$)

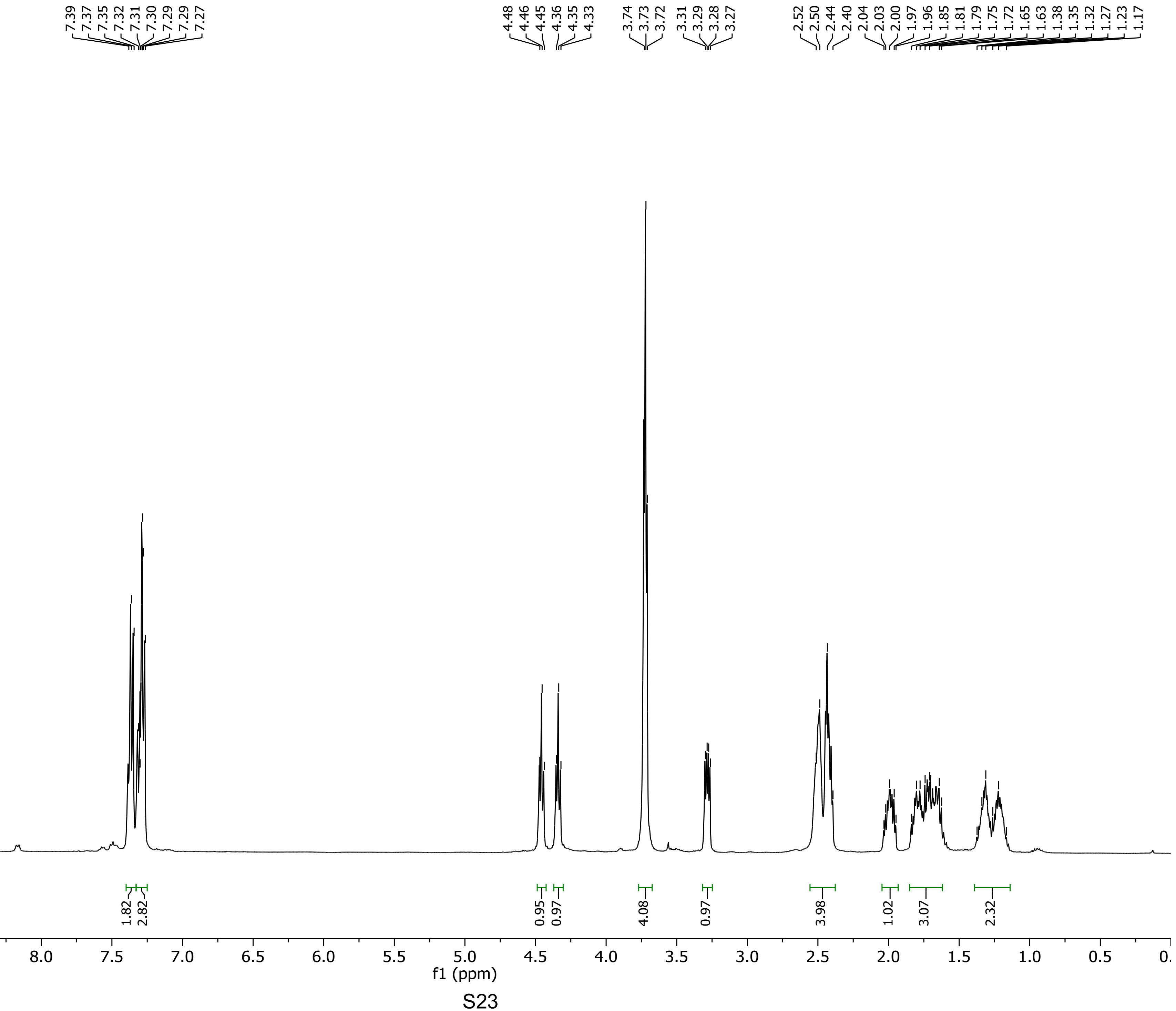

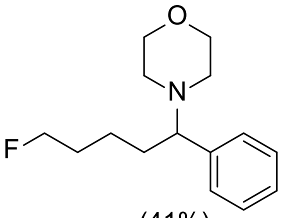

5eba (41%)

$^{13}C\{^1H\}$ NMR (100 MHz / $CDCl_3$)

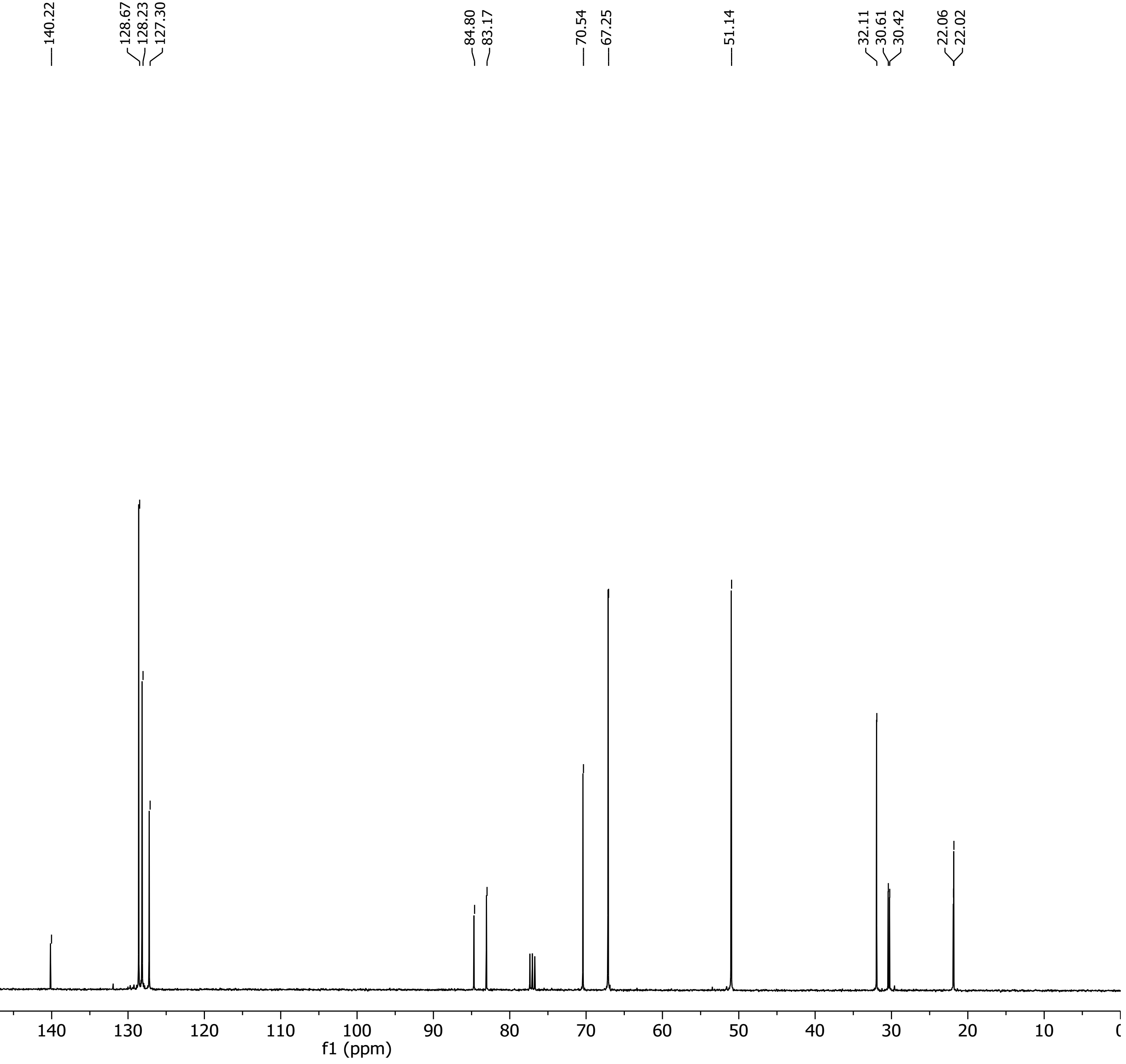

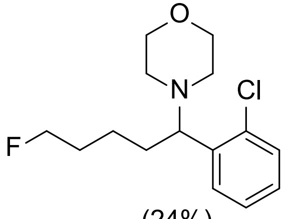

5ebe (24%)

^{1}H NMR (400 MHz / $CDCl_3$)

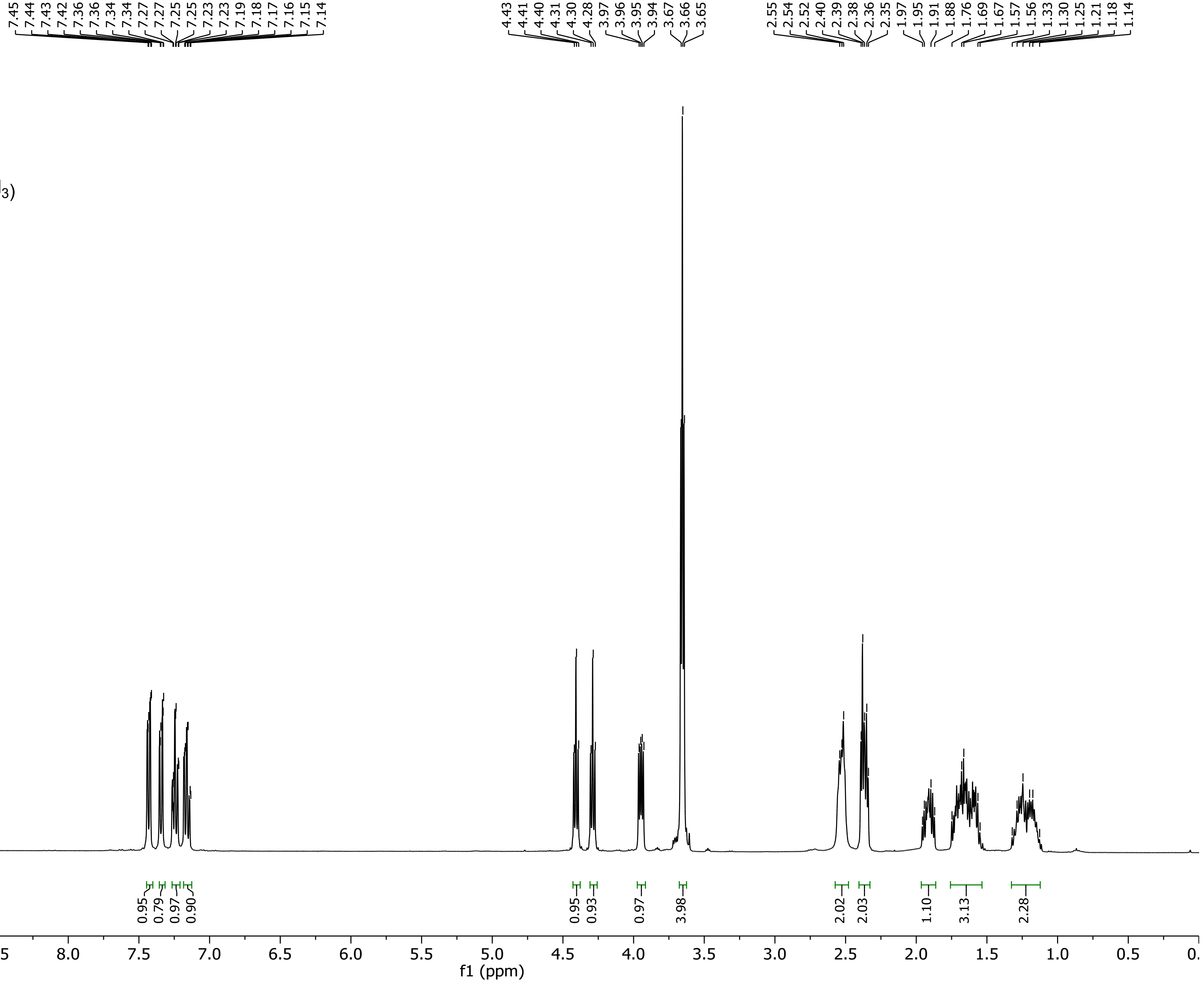

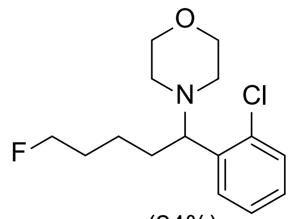

5ebe (24%)

$^{13}C\{^{1}H\}$ NMR (100 MHz / $CDCl_3$)

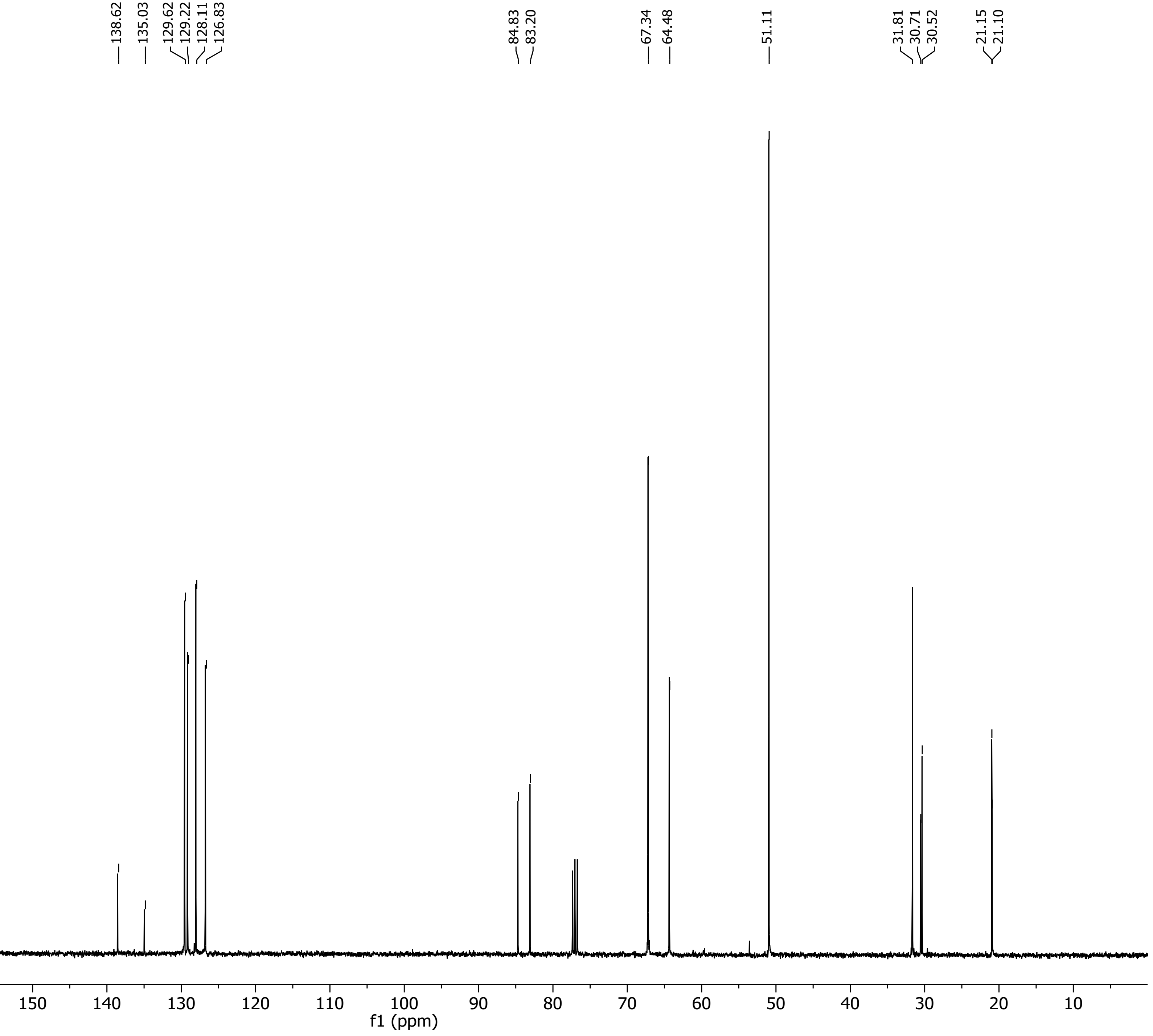

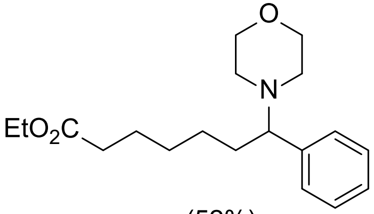

(52%)

5gba

^{1}H NMR (400 MHz / $CDCl_3$)

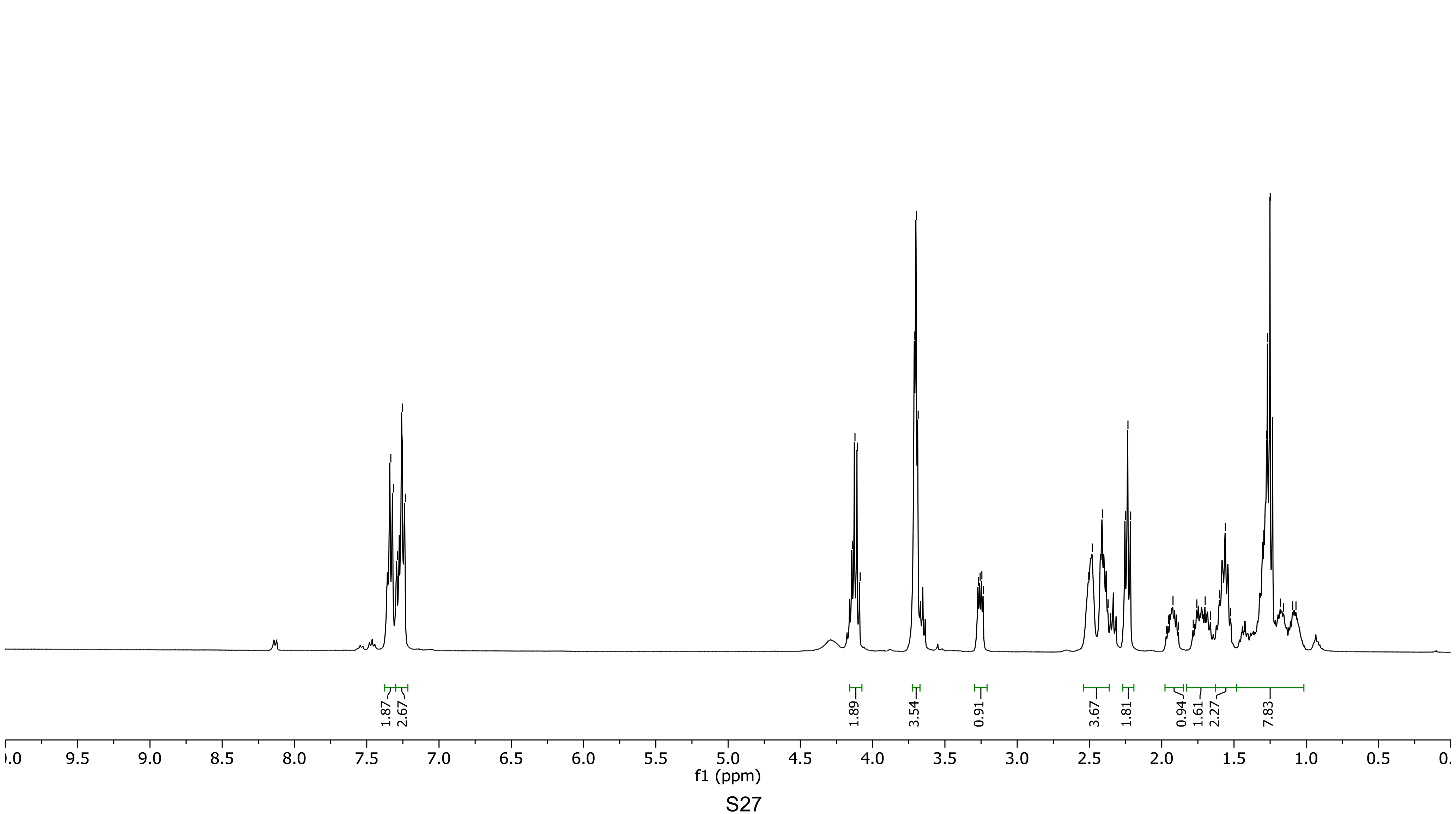

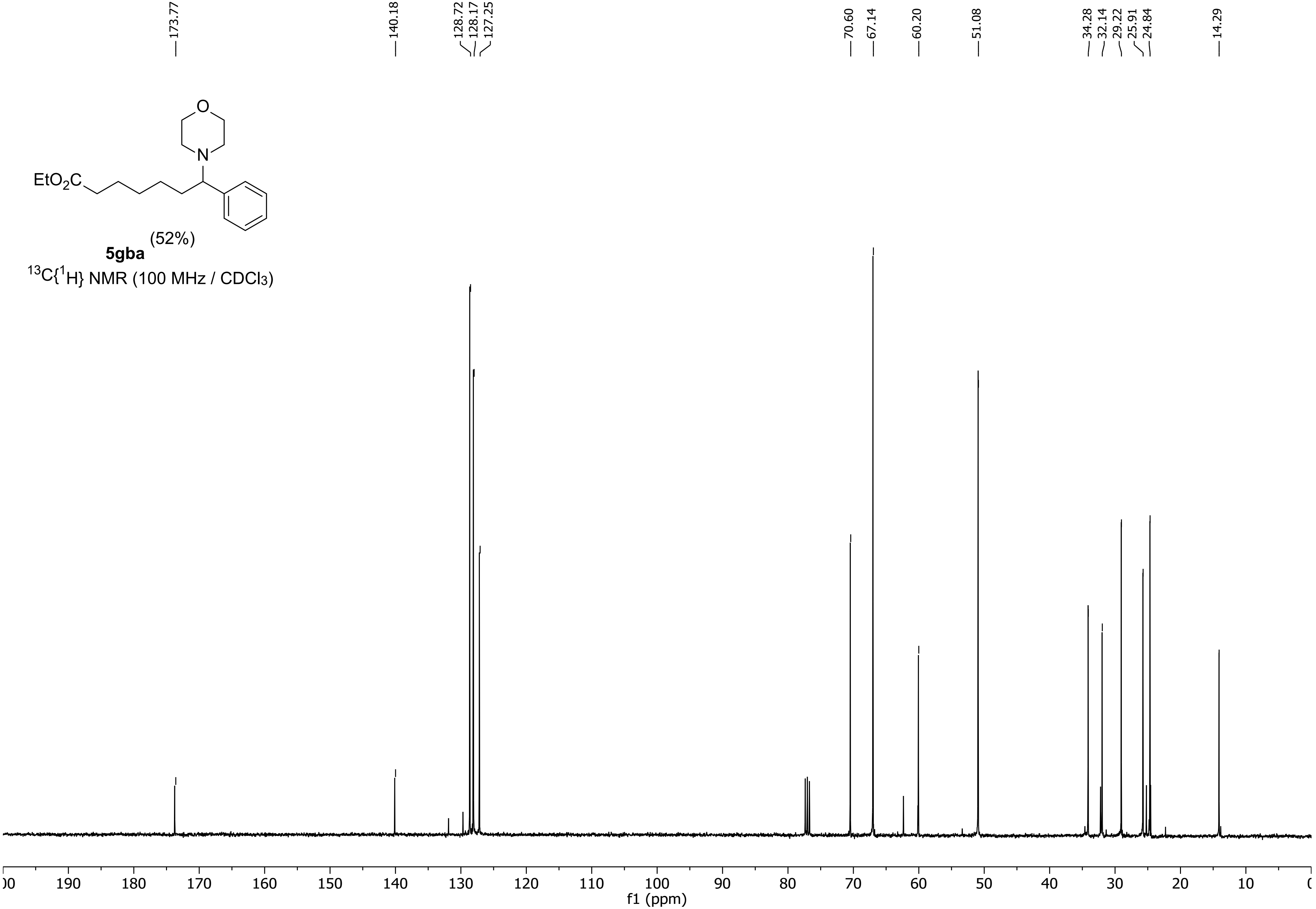
173.77
140.18
128.72
128.17
127.25
70.60
67.14
60.20
51.08
34.28
32.14
29.22
25.91
24.84
14.29
O
N
EtO2C
(52%)
5gba
13C{1H} NMR (100 MHz / CDCl3)
190
180
170
160
150
140
130
120
110
100
90
80
70
60
50
40
30
20
10
f1 (ppm)

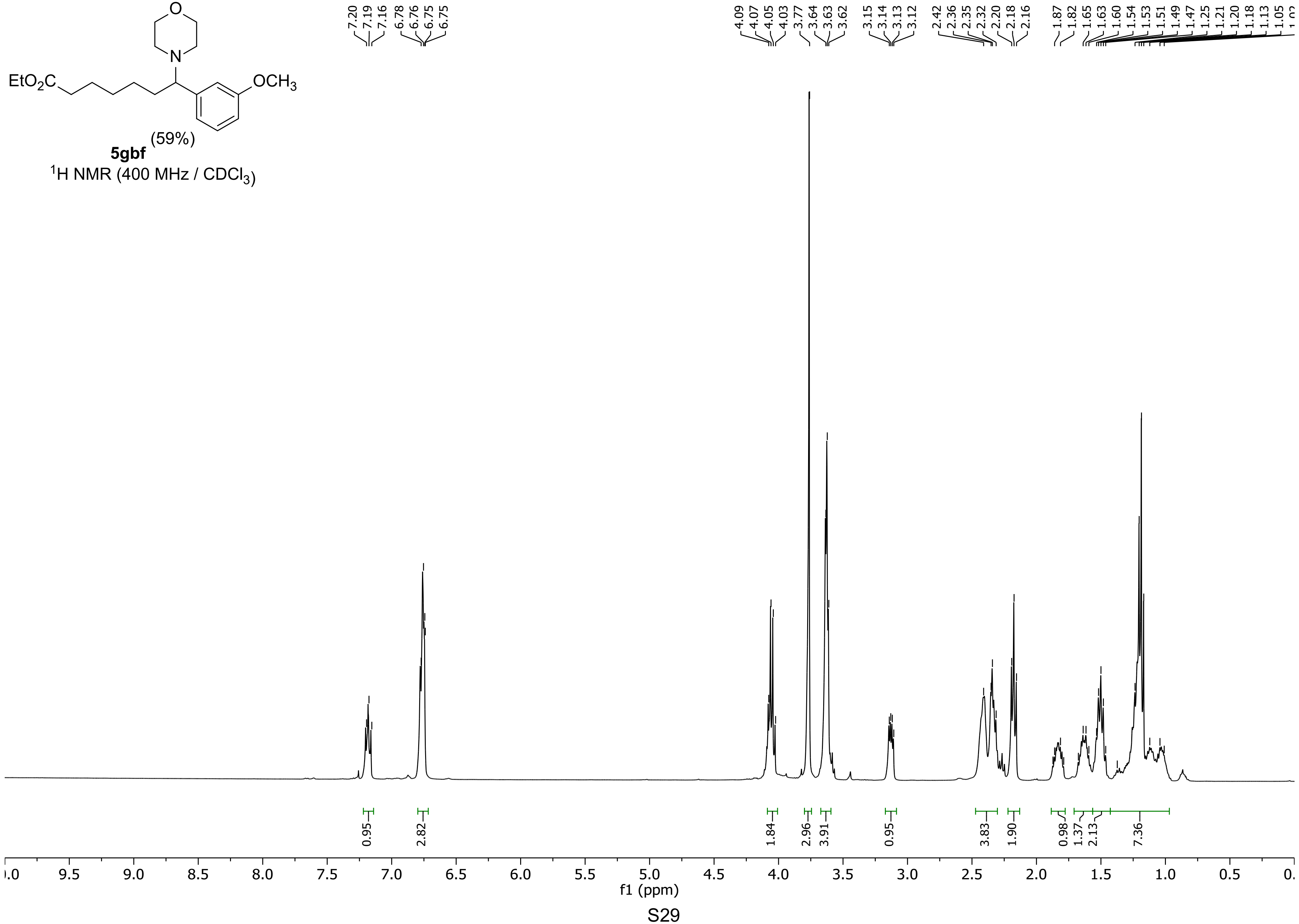
EtO2C
OCH3
O
N
(59%)
5gbf
1H NMR (400 MHz / CDCl3)
f1 (ppm)

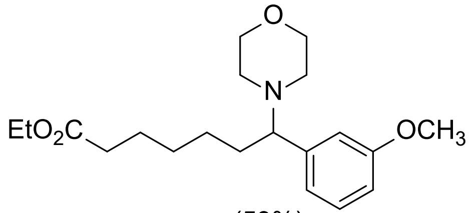

5gbf (59%)

$^{13}C\{^{1}H\}$ NMR (100 MHz / $CDCl_3$)

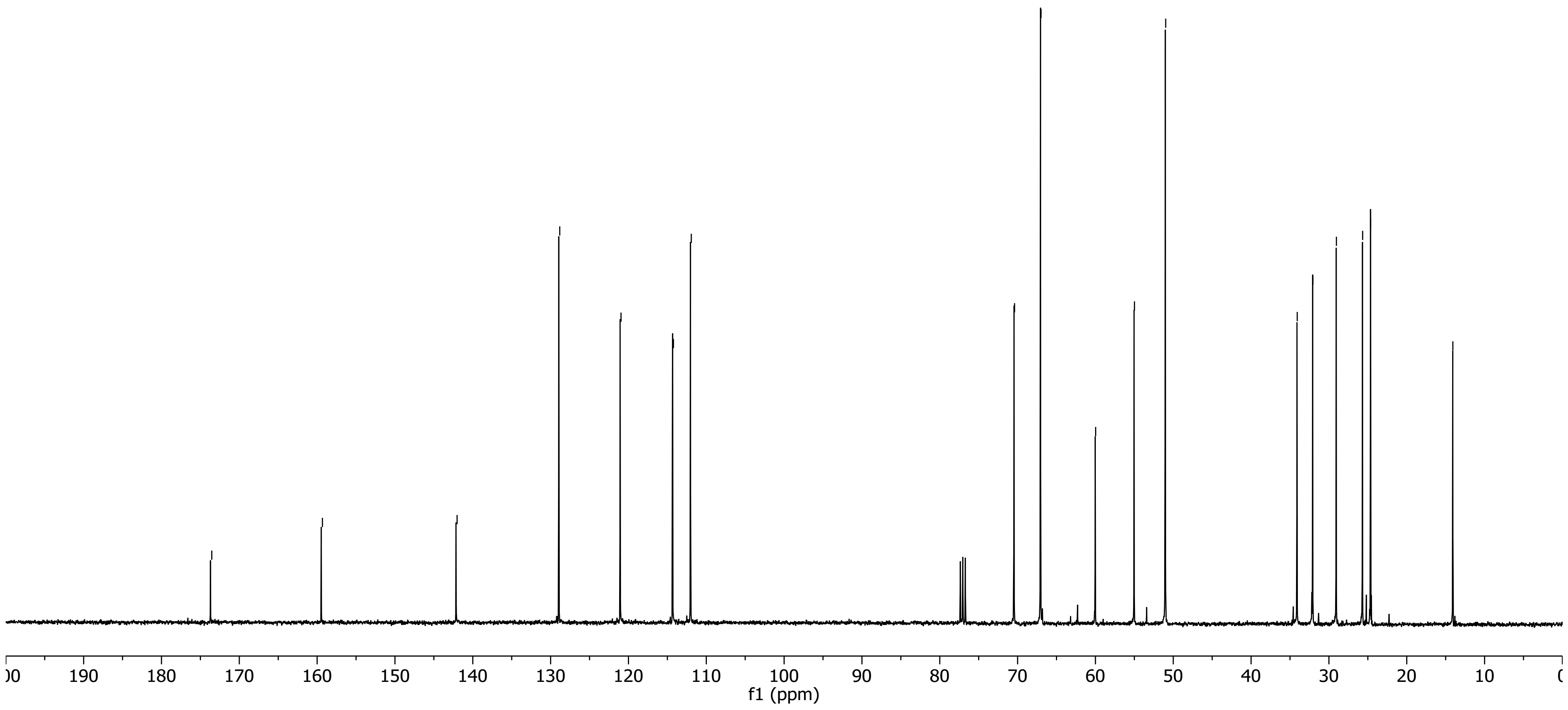

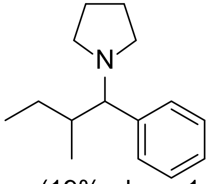

5ida (19%, d.r. = 1:1)

^{1}H NMR (400 MHz / $CDCl_3$)

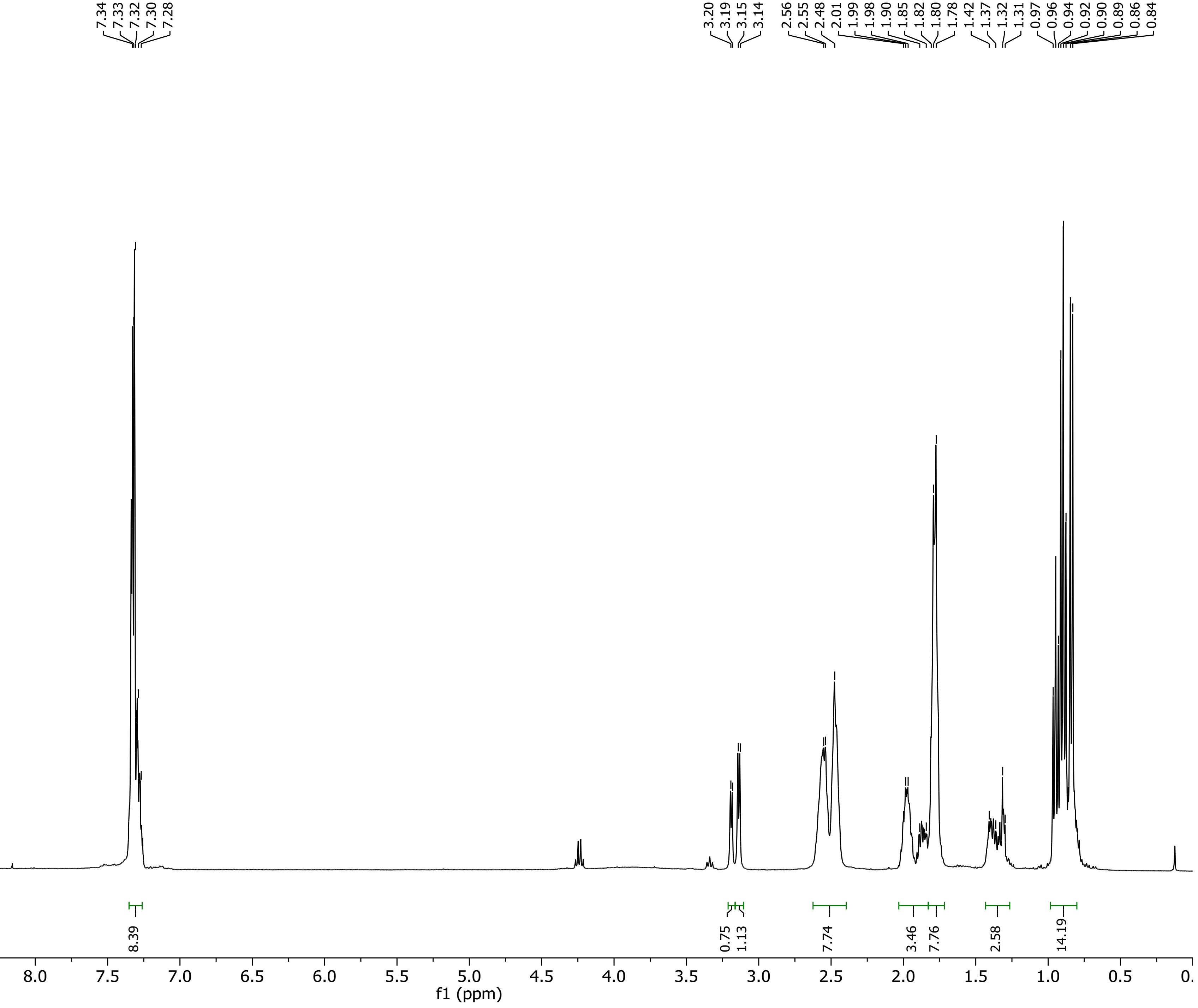

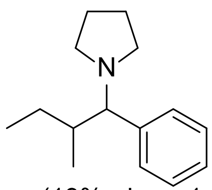

5ida (19%, d.r. = 1:1)

$^{13}C\{^{1}H\}$ NMR (100 MHz / $CDCl_3$)

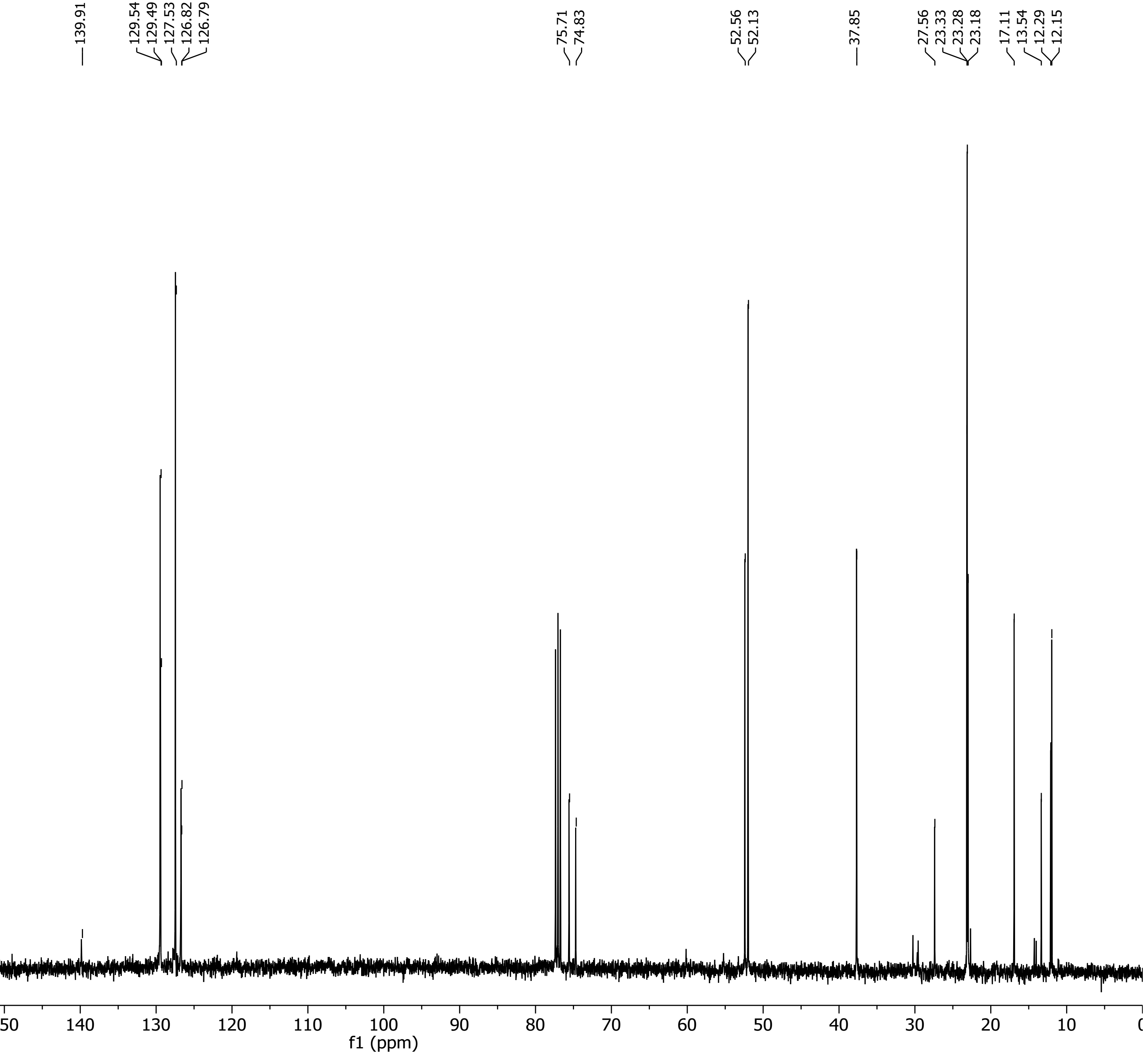

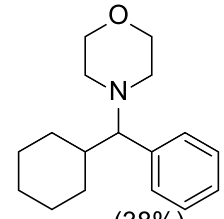

(38%)

5jba

^1H NMR (400 MHz / $CDCl_3$)

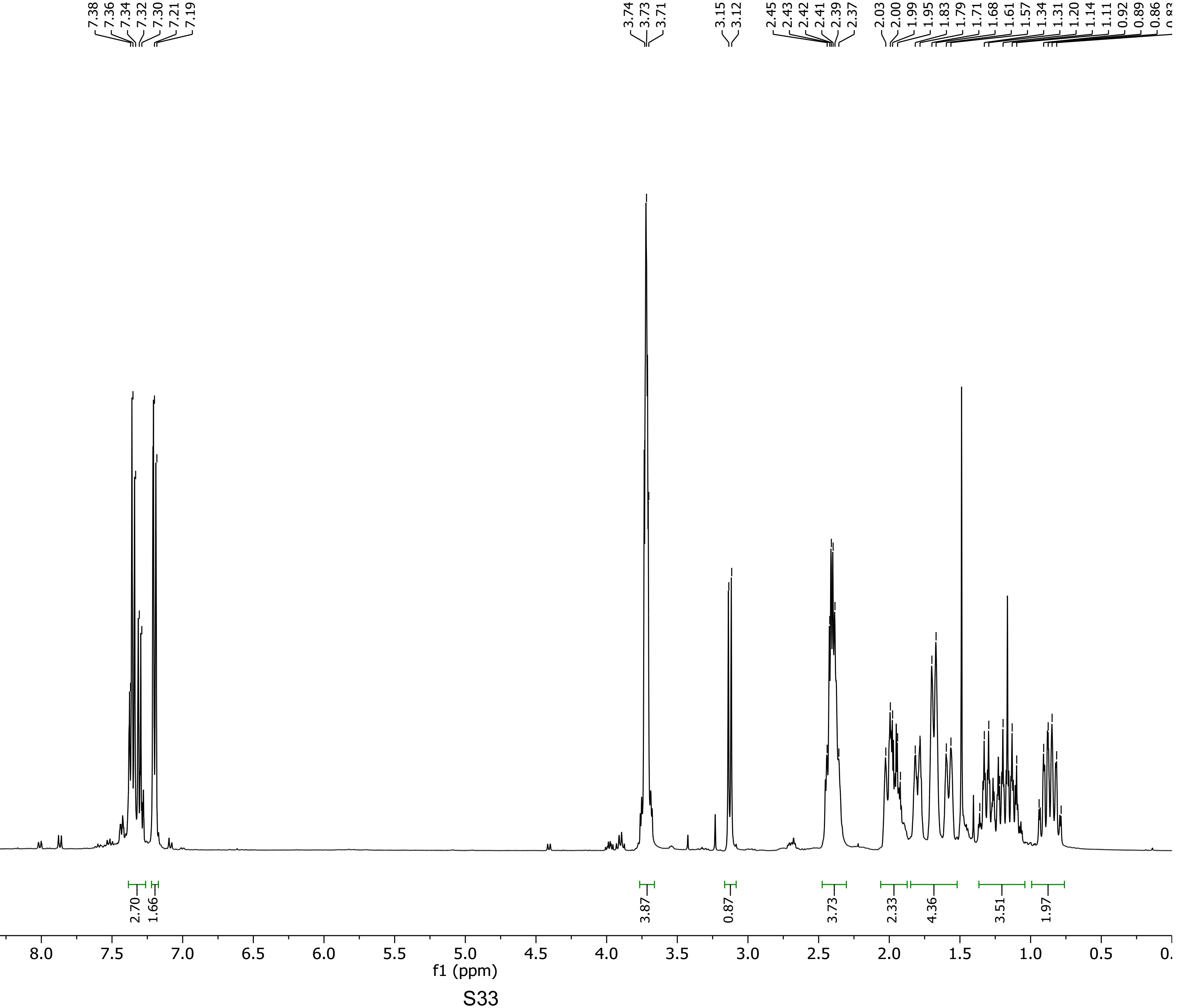

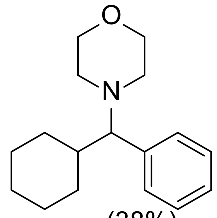

(38%)

5jba

$^{13}C\{^{1}H\}$ NMR (100 MHz / $CDCl_3$)

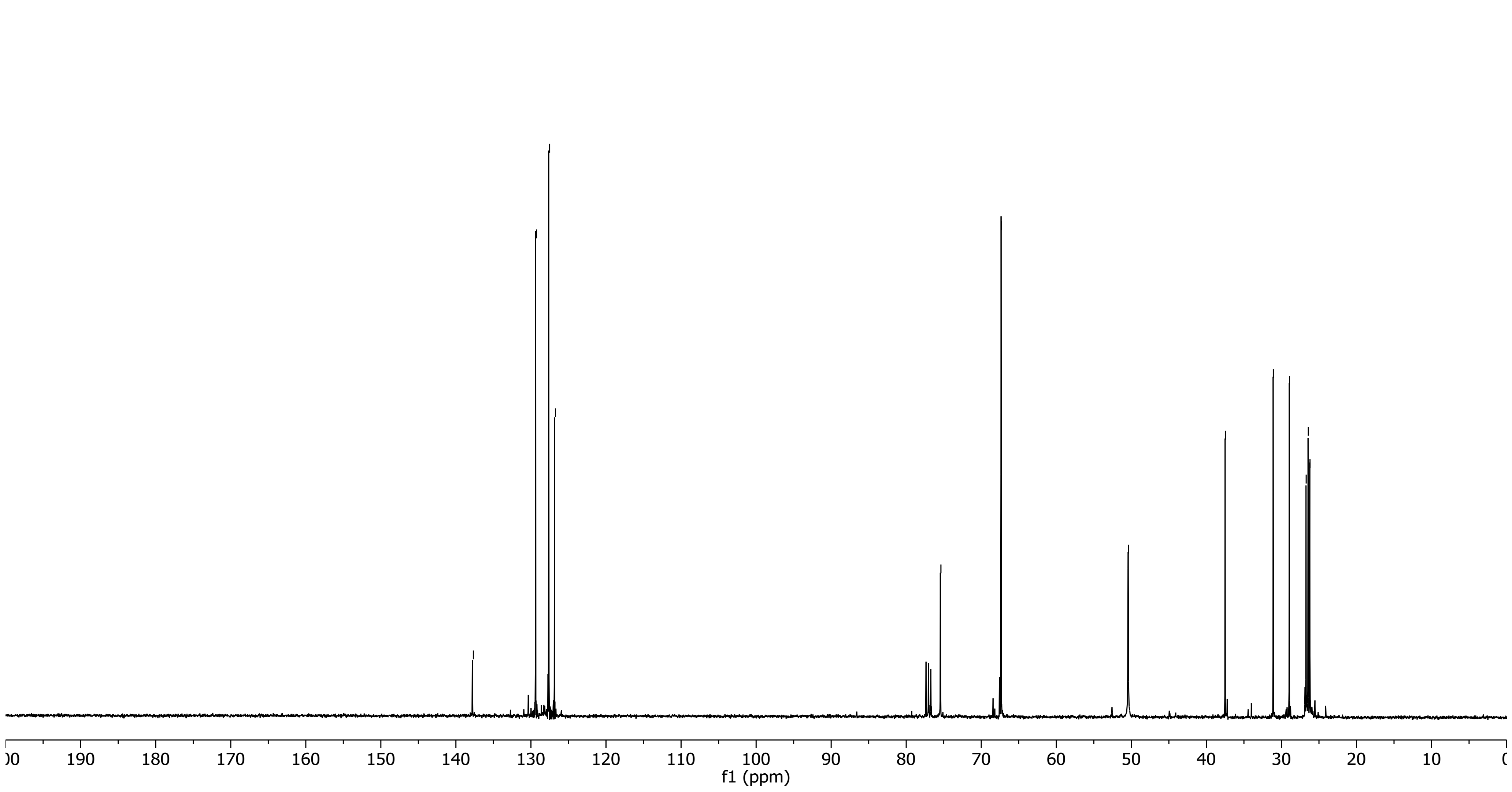

Supplement: File 1 — Experimental procedures, compound characterization data, and NMR spectra for all compounds. [file Beilstein_J_Org_Chem-20-2834-s001.pdf]
